# Targeting PD-1^+^ T cells with chimeric antigen receptors to reduce the HIV reservoir

**DOI:** 10.1126/sciadv.aeb7602

**Published:** 2026-04-24

**Authors:** Laura Ermellino, Riddhima Banga, Spiros Georgakis, Nicole P. Kadzioch, Francesco Procopio, Ana Alcaraz-Serna, Oscar Alfageme-Abello, Raphaël Porret, Rebecca Cecchin, Michail Orfanakis, Rachel Schelling, Cloé Brenna, Duy-Cat Can, Mathilde Foglierini, Oliver Y. Chén, Laurent Perez, Craig Fenwick, Matthieu Perreau, Constantinos Petrovas, Roberto F. Speck, Giuseppe Pantaleo, Yannick D. Muller

**Affiliations:** ^1^ Division of Immunology and Allergy, Lausanne University Hospital and University of Lausanne, Lausanne, Switzerland.; ^2^ Institute of Pathology, Department of Laboratory Medicine and Pathology, Lausanne University Hospital and University of Lausanne, Lausanne, Switzerland.; ^3^ Department of Infectious Diseases and Hospital Epidemiology, University Hospital of Zurich, University of Zurich, Zurich, Switzerland.; ^4^ Bioinformatics Platform, Department of Laboratory Medicine and Pathology, Lausanne University Hospital and University of Lausanne, Lausanne, Switzerland.

## Abstract

The unique ability of chimeric antigen receptor (CAR) T cells to infiltrate tissues is revolutionizing our perspectives for tackling severe, refractory and otherwise untreatable diseases. In HIV, CAR-T cells have been designed to target viral biomarkers, with limited success so far. Here, we investigated the possibility of redirecting CAR-T cells against a cellular biomarker of the HIV reservoir, the programmed cell death protein 1 (PD-1). We designed two second-generation 4-1BB-CARs using the scFv of either a blocking (bPD1-CAR) or a nonblocking (nbPD1-CAR) anti–PD-1 monoclonal antibody. The CAR avidity modulated T cell sensitivity, trogocytosis, and effector functions, independently of the PD-1 signaling domain. Both anti–PD-1 CAR-T cells could persist for 70 days in HIV-infected humanized mice, correlating with viral protection and a disruption of the lymphoid architecture in the white pulp of the spleen. Together, our results open strategic avenues for reducing the HIV reservoir by demonstrating the feasibility of depleting specific T cell subpopulations.

## Teaser

CAR T cells can be engineered against PD-1, a nonvirological biomarker of the HIV reservoir.

## INTRODUCTION

Chimeric antigen receptor (CAR) T cells are revolutionizing strategic therapeutic approaches for tackling severe, refractory and otherwise untreatable diseases. Beyond the success of CAR T cells in oncological conditions (*1*), the past years have seen the emergence of cellular approaches to treat systemic lupus erythematosus, idiopathic inflammatory myositis, systemic sclerosis, but also neurological and other autoimmune conditions (*2*–*4*). The unexpected results of long-term remission in patients who otherwise require chronic immunosuppression have led to the concept of immune resetting (*5*). Thus, CAR-T cells have unique abilities to clear the autoreactive B cell repertoire within tissues and lymphoid organs, where monoclonal antibody–based therapies (mAbs) have failed (*6*).

The first clinical trials using engineered T cells were done in the field of HIV in the early nineties (*7*). Conceptually, T cells were redirected against the HIV envelope, taking advantage of the fact that HIV-infected cells can be detected because they express virus-specific biomarkers at their cell surface (*8*). Thus, T cells have been first engineered with a CD4 extracellular domain fused to a CD3ζ signaling domain to kill HIV-infected cells (*9*, *10*). Since the emergence of broadly neutralizing antibodies (*11*, *12*), T cells have been armed with single-chain variable fragments (scFvs) targeting several regions of the HIV envelope in addition to the CD4 binding site such as the V1V2 apex or the membrane-proximal external region (MPER) of gp41 glycan site. Although no benefit has been reported in CAR-T–treated HIV-infected patients to date, several clinical trials are ongoing evaluating dual/bispecific anti-HIV CARs (*13*).

Over the last two decades, much effort has been used to understand the complexity of the HIV reservoir to foster the development of therapeutic interventions that could lead to a functional cure for HIV. Several subsets of CD4^+^ T cells harbor transcriptionally silent proviruses at different frequencies and can escape immune surveillance (*14*). In particular, there is a viral enrichment within the lymph nodes of aviremic individuals treated with antiretroviral therapies (ARTs) in CD4^+^ T cells expressing the programmed cell death protein 1 (PD-1) receptor, namely follicular helper cells (T_FH_s) (*15*, *16*). Others found elevated levels of integrated HIV DNA in circulating memory PD-1^+^ CD4^+^ T cells (*16*, *17*). Last, PD-1 has also been described as a marker for HIV persistence in other anatomical sites such as rectal tissues (*18*).

Considering the unique abilities of T cells to migrate deep into the tissues, we hypothesize that T cells can be redirected against the cellular marker PD-1, which could contribute to reduce the HIV reservoir. Recent work by Eichholz *et al.* (*19*), has explored PD-1 as a potential target for HIV reservoir reduction in simian immunodeficiency virus (SIV)–infected rhesus macaques using CAR-T cells engineered with pembrolizumab- or nivolumab-derived scFvs. T cells transduced with those anti–PD-1-CARs effectively depleted SIV-infected CD4^+^ T cells in germinal centers (*19*).

Here, we developed second-generation 4-1BB CARs using scFv derived from two other anti–PD-1 antibodies discovered by our group (*20*, *21*). These were named blocking (b), as one targets the PD-1 binding site for PD-L1/PD-L2, and nonblocking (nb), as the other recognizes a noncompetitive, allosteric epitope on PD-1. The main objective was not to prevent PD-L1/2 from binding to PD-1 but to study the functional CAR T cell activities against two distinct epitopes on PD-1. We showed that the functional activity of engineered T cells is modulated by the CAR-binding epitope rather than its affinity. Both CAR-T cells were detected following adoptive cell transfer (ACT) correlating with reduced number of PD-1^+^ cells, delayed HIV rebound and loss of the lymphoid architecture in the white pulp of the spleen of humanized mice. These findings underscore the potential of anti–PD-1 CAR-T cells in resetting the lymphoid architecture and thereby reducing the size of the HIV reservoir.

## RESULTS

### Blocking and nonblocking anti–PD-1 antibodies characterization and CAR design

**Fig. 1.**
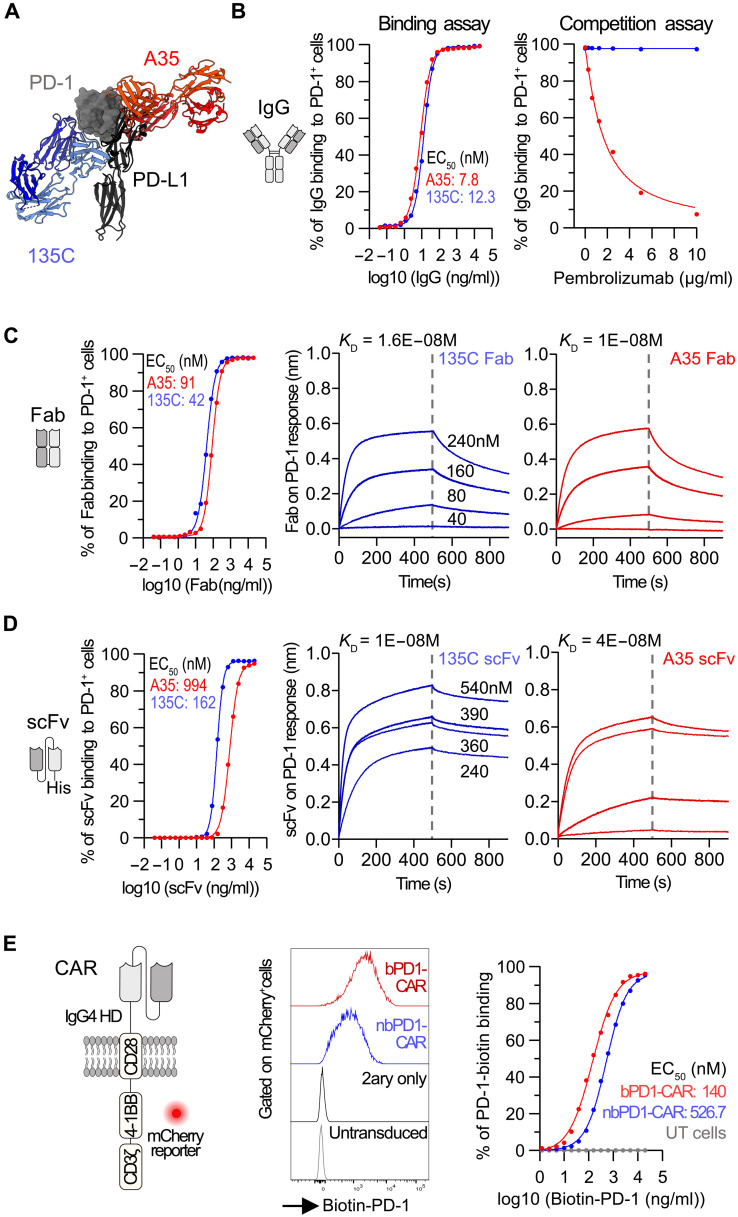
PD-1 binding to CAR-T cells is epitope dependent and not necessarily predicted by the antibody affinity. (**A**) In silico model representing the binding of Fab fragment from clones A35795 (A35) and 135c139d6 (135c) compared to the hPD-1/hPD-L1 complex (PDB ID: 4ZQK). (**B**) Binding of A35 and 135c IgG to human PD-1 (left). Competitive binding assay with pembrolizumab and biotin-labeled A35 IgG or 135c IgG (right). Symbols are means of three (left) or two (right) independent experiments. (**C**) Binding of A35 and 135c Fabs to human PD-1 (left). Biolayer interferometry of Fabs–PD-1 interactions (right). Symbols are means of three independent experiments (left). (**D**) Binding of A35 and 135c scFvs-His to human PD-1 (left). Biolayer interferometry of scFvs–PD-1 interactions (right). Symbols are the mean of three independent experiments (left). (**E**) Second-generation CAR design (left). Biotinylated PD-1 binding to anti–PD-1 CARs. Representative flow cytometry and mean of four independent experiments. PD-1, programmed cell death protein 1; PD-L1, programmed cell death ligand 1; IgG, immunoglobulin G; Fab, fragment antigen-binding region; *K*_D_, equilibrium dissociation constant; scFv, single-chain variable fragment; His, histidine; CAR, chimeric antigen receptor; EGFRt, truncated epidermal growth factor receptor; HD, hinge domain; bPD1-CAR, blocking anti–PD-1 CAR; nbPD1-CAR, nonblocking anti–PD-1 CAR; UT, untransduced cells.

We selected the scFv of two anti–PD-1 monoclonal antibodies previously discovered by our center, interacting with two different regions of the PD-1 receptor: clone A35795 (A35) blocking the interaction with PD-L1 and clone 135c139d6 (135C) binding to a different epitope independently of the PD-1/PD-L1 engagement site (Fig. 1A) (*20*, *21*). We produced immunoglobulin G1 (IgG1) antibodies and confirmed their binding to Jurkat cells overexpressing PD-1 (Fig. 1B). A competitive binding assay with increasing concentrations of anti–PD-1 monoclonal antibodies pembrolizumab or nivolumab demonstrated the blocking and nonblocking nature of clones A35 and 135C, respectively (Fig. 1B and fig. S1A). To further evaluate the affinity of both clones, we produced them as fragment antigen-binding domains (Fab) and scFv. Both Fabs had a similar dissociation constant (*K*_D_) in the range of 10 to 16 nM, while clone 135C, when expressed as scFv, exhibited a four times lower *K*_D_ than clone A35 (Fig. 1, C and D). We next engineered human T cells expressing both scFvs as a 4-1BB second-generation CAR flanked to a ribosomal skipping motif and mCherry reporter. In contrast to the results obtained with purified scFv, the binding of biotinylated PD-1 was lower for the 135C CAR, suggesting a lower avidity (Fig. 1E and fig. S1B).

### Anti–PD-1 CARs cytotoxic activity is scFv dependent

**Fig. 2.**
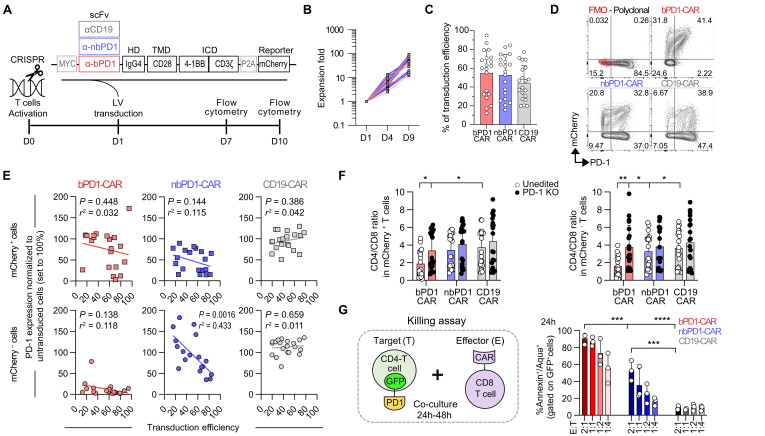
The nonblocking anti–PD-1 CAR showed intermediate functional activities in vitro. (**A**) Experimental design for engineering human anti–PD-1 CAR-T cells. (**B**) Expansion of primary anti–PD-1 CAR-T cells compared to the anti-CD19 CAR control (*n* = 7 donors, seven independent experiments). (**C**) Cumulative data of transduction efficiency for donors reported in (D) and (E). The mean ± SD values eight donors, eight independent experiments, two to three replicates transduced with different MOI is shown. Two-way ANOVA, Tukey’s multiple comparison test. (**D**) Representative flow cytometry plots showing mCherry and PD-1 expression 6 days after transduction (gated on CD3^+^ living cells). (**E**) Correlation between PD-1 expression and the percentage of transduction efficiency. Cherry-positive and Cherry-negative population are shown. For each experiment the percentage of PD-1^+^ cells were assessed in Cherry-negative and Cherry-positive cells and normalized to PD-1 expression in untransduced T cells (set as 100%). Eight donors, eight independent experiments each with two to three internal replicates (with different MOI transduction: 1.4 ± 0.6). Simple linear regression tests. (**F**) CD8/CD4 ratio in mCherry ± T cells comparing anti–PD-1 CAR and CD19 CAR-T cells ± PD-1 knockout. Data are presented as mean ± SD values of eight donors, eight independent experiments, two to three internal replicates). Two-way ANOVA, Tukey’s multiple comparisons test. (**G**) Schematic representing the killing assay (left). Cumulative percentage of annexin/Aqua^+^ CD4^+^PD-1^+^GFP^+^ target cells after 24 hours of coculture (right). Mean ± SD of three donors and three independent experiments is shown. Two-way ANOVA, Tukey’s multiple comparison test. Only statistical differences are reported as follows: *P ≤ 0.05, **P ≤ 0.01, ***P ≤ 0.001, and ****P ≤ 0.0001. HD, hinge domain; TMD, transmembrane domain; ICD, intracellular domain; LV, lentivirus; scFv, single-chain variable fragment; CAR, chimeric antigen receptor; bPD1-CAR, blocking anti–PD-1 CAR; nbPD1-CAR, nonblocking anti–PD-1 CAR; FMO, fluorescence minus one; UT, untransduced; KO, knockout.

To further evaluate the functional activity of both CARs in primary T cells, we included an anti-CD19 CAR control in addition to the nonblocking (nbPD1, 135C) and blocking (bPD1, A35) CARs. All CARs had a Myc tag at the N terminus and expressed mCherry as a reporter (Fig. 2A). We obtained comparable surface expression, transduction efficiencies, and expansion rates of all three CARs (Fig. 2, B and C, and fig. S2). Notably, we found a spontaneous and significant loss of PD-1^+^ cells, mainly in the Cherry^−^ population of the bPD1-CAR condition (Fig. 2D and fig. S3A). Such depletion was also present in the nbPD1-CAR condition but only at a high transduction level (Fig. 2E and fig. S3A). The PD-1 loss found with the bPD1-CAR correlated with a significant reduction in the number of CD4^+^ T cells, likely because CD8^+^ T cells expressed lower levels of PD-1 during in vitro expansion (Fig. 2F, fig. S3B). When performing a knockout of PD-1 (day 0) before CAR editing (day 1), we could prevent the loss of CD4^+^ T cells (Fig. 2F and fig. S3C), confirming the hypothesis that CD4^+^ T cells loss was related to their PD-1 surface expression.

To evaluate the cytotoxicity of both CARs, we next expanded separately human CD8^+^ CAR-T cells from CD4^+^ T cells, which were transduced with a PD-1–green fluorescent protein (GFP) fusion protein to ensure stable PD-1 expression (fig. S4, A and B). The CD4^+^ T cells were killed by both CARs, the bPD1-CAR being more efficient (fig. S4, C and D, and Fig. 2G).

### bPD1-CAR-T cells show enhanced sensitivity and cytotoxicity independently of the PD-1 signaling domain

**Fig. 3.**
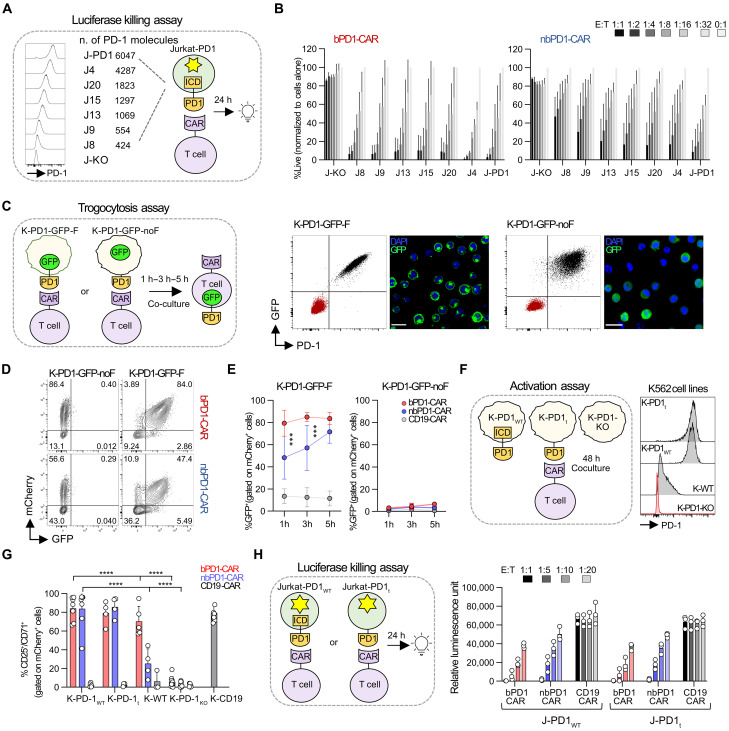
PD-1 trogocytosis is CAR dependent and does not inhibit T cells activation. (**A**) Generation of PD-1-transgenic luciferase^+^ Jurkat cells with increasing PD-1 molecules number. (**B**) Luciferase-based killing assay of blocking and nonblocking CAR-T cells. Mean ± SD of three donors and three independent experiments is shown. (**C**) Phenotype of the K562 cell lines expressing either a PD-1-GFP-F fusion protein (K-PD-1-GFP-F) or PD-1 and GFP proteins with a ribosomal skipping motif in-between (K-PD-1-GFP-noF) used for the trogocytosis assay. Scale bars, 25 μm. (**D**) Representative flow cytometry 1 hour after coculture of K-PD-1-GFP-F or -PD-1-GFP-noF with anti–PD-1 CAR-T cells. (**E**) Cumulative data showing GFP expression in CAR-T cells over time. Mean ± SD values of six donors and four independent experiments is shown. Two-way ANOVA, Tukey’s multiple comparison test. (**F**) Phenotype of the K562 cell lines expressing either the wild-type PD-1 (K-PD-1_WT_) or a truncated PD-1 lacking (K-PD-1_t_) the intracellular domain used for the activation assay. (**G**) Cumulative percentage of CD25^+^/CD71^+^ CAR-T cells after a 48 hours coculture with K-PD-1_WT_ or K-PD-1_t_. Mean ± SD values of four to eight donors and seven independent experiments is shown. Two-way ANOVA, Tukey’s multiple comparison test. (**H**) Luciferase killing assay using PD-1_WT_ or PD-1_t_ luciferase^+^ Jurkat cells as targets. Mean ± SD values of three donors, three independent experiments is shown. Two-way ANOVA, Tukey’s multiple comparison test was used to compare the same condition in JPD-1 versus JPD-1_t_. Only statistical differences are reported as follows: *P ≤ 0.05, **P ≤ 0.01, ***P ≤ 0.001, and ****P ≤ 0.0001. JPD-1, Jurkat-PD-1; CAR, chimeric antigen receptor; bPD1-CAR, blocking anti–PD-1 CAR; nbPD1-CAR, nonblocking anti–PD-1 CAR; E:T, effector:target; K-PD-1-GFP-F, K562 PD-1-GFP-F fusion protein; K-PD-1-GFP-noF, K562 PD-1^+^ GFP^+^; K-PD-1, K562 PD-1^+^; K-PD-1-t, K562 PD-1 truncated; K-PD-1-KO, K562 PD-1 KO; JPD-1-t, Jurkat PD-1 truncated.

To better characterize the functional activity of anti–PD-1 CARs, we next assessed their sensitivity and PD-1 trogocytosis capacities. Starting from a luciferase positive PD-1 knockout (KO) Jurkat cell line, we generated seven clones with increasing levels of PD-1 ranging from 424 to 6047 PD-1 molecules and used them as targets in a bioluminescent killing assay (Fig. 3A). To further reduce the potential off-target activities we coedited *TRBC* (T cell receptor β chain) in addition to the endogenous PD-1 receptor of primary T cells (fig. S5, A to C). Consistently with the in vitro killing results in primary cells, bPD1-CAR T cells exhibited higher sensitivity with low expressing cell lines as well (Fig. 3B). Secretion of key effector molecules [interferon-γ (IFN-γ), tumor necrosis factor–α (TNF-α), and interleukin-2 (IL-2)] and cytolytic protein expression [perforin and granzyme B (Grzb)] was next evaluated. The results confirmed the higher sensitivity of the bPD1-CAR against wild type K562 cells but not PD-1 high transgenic target cells (fig. S6). As expected, CD4 produced higher levels of TNF-α and IL-2, while the CD8 population is more skewed toward IFN-γ secretion. For the trogocytosis assay, we transduced K562 PD-1 KO cells with a lentiviral vector coding for GFP-only or a PD-1-GFP fusion protein (Fig. 3C). In line with the sensitivity results, the bPD1-CAR acquired GFP-PD-1 more efficiently than the nbPD1-CAR (Fig. 3, D and E).

Given that the intracellular domain (ICD) of PD-1 mediates inhibitory signaling and the CARs ability to uptake PD-1, we next evaluated the contribution of the PD-1-ICD to CAR-T cell functional activity. We engineered three K562 cell lines expressing PD-1 either with a truncated ICD (PD-1_t_) or the wild type molecule (PD-1_WT_) and compared them to a WT K562 cell line spontaneously expressing low levels of PD-1 (Fig. 3F). We did not observe any differences in the level of activation or in the cytotoxicity between the truncated and WT PD-1 for both PD-1^high^ transgenic cell lines, suggesting that the uptake of PD-1 has no direct impact on CAR-T cell function (Fig. 3, G and H).

### T cells exhaustion and terminal differentiation are anti–PD-1 CAR dependent

**Fig. 4.**
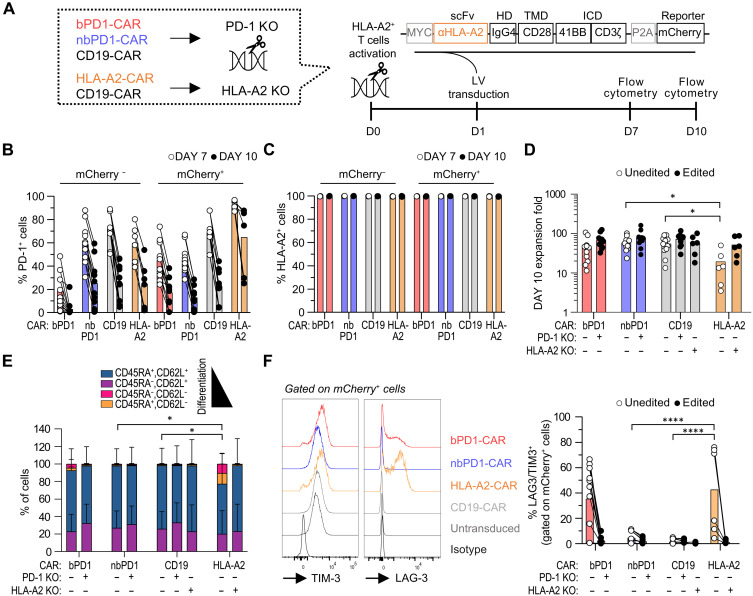
Constitutive expression of endogenous receptors and CAR-T cells sensitivity modulate terminal differentiation, exhaustion, and cell death. (**A**) Experimental design. HLA-A2^+^ human T cells were isolated and activated ± prior editing of PD-1 and/or HLA-A2. (**B**) PD-1 expression in mCherry^+^ and mCherry^−^ cells on days 7 and 10. Mean of three to five donors in three to five independent experiments with one to two replicates transduced at different MOI (1.4 ± 0.6) for each independent experiments. (**C**) HLA-A2 expression in mCherry^+^ and mCherry^−^ cells on day 7 and 10. Mean of two donors in two independent experiments with one to two internal replicates (different MOI transduction: 1.4 ± 0.6). (**D**) Expansion folds of unedited versus edited CAR-T cells on day 10. Mean ± SD values of three to five donors in three to five independent experiments with one to two internal replicates (different MOI transduction: 1.4 ± 0.6). Statistics: Two-way ANOVA, Dunnett’s multiple comparison test was performed comparing each condition with the corresponding HLA-A2 CAR condition. (**E**) Cumulative data showing CD62L/CD45RA expression of mCherry^+^ cells in edited versus unedited condition for PD-1 or HLA-A2. The mean ± SD values of three to five donors in three to five independent experiments with one to two internal replicates (different MOI transduction 1.4 ± 0.6) are shown. Kruskal-Wallis and Dunn’s multiple comparison test was performed comparing each condition with the corresponding HLA-A2 CAR condition. (**F**) Representative flow cytometry showing TIM-3 and LAG-3 expression in the different CAR populations on day 10 (left). Cumulative percentage of LAG-3^+^/TIM-3^+^ double-positive cells (right). Paired data of three to five donors in three to five independent experiments with one to two internal replicates (different MOI transduction: 1.4 ± 0.6) are shown. Two-way ANOVA, Dunnett’s multiple comparison test was performed comparing each condition with the corresponding HLA-A2 CAR condition. Only statistical differences are reported as follows: *P ≤ 0.05, **P ≤ 0.01, ***P ≤ 0.001, and ****P ≤ 0.0001.

Since anti–PD-1 CAR-T cells naturally upregulate PD-1 upon activation and can uptake external PD-1 by trogocytosis, we next evaluated whether this could contribute to repetitive stimulation ultimately accelerating T cell differentiation and exhaustion. As a positive control, we engineered HLA-A2^+^ T cells with an anti–HLA-A2 (A2) CAR using a previously reported scFv (*22*), and as negative controls, we deleted either HLA-A2 or PD-1 from the A2 or PD1–CAR-T cells, respectively (Fig. 4A and fig. S7, A and B). While for the bPD1 CAR condition, PD-1 expression was strongly reduced, for the HLA-A2 CAR condition, HLA-A2 remained highly expressed in both cherry positive and negative cells (Fig. 4, B and C). Consequently, HLA-A2 CAR-T showed significantly reduced expansion and a terminally differentiated profile, as indicated by the expression of exhaustion markers (LAG-3/TIM-3). This phenotype was restored by editing HLA-A2 (Fig. 4, D to F, and fig. S7, C to F). To a lesser extent, the bPD1-CAR-T cells also showed higher levels of exhaustion and terminal differentiation, which was not the case for the nbPD1-CAR and the CD19-CAR control conditions. Together, these results suggest that the inducible nature of PD-1 can preserve anti–PD-1 CAR-T cells from early exhaustion in a CAR-avidity–dependent manner.

To better assess the contribution of cis and trans presentation of PD-1, we next diluted 100 unedited anti–PD-1 and anti–HLA-A2 CAR T cells into 2.5, 5, and 10 ml of medium between days 3 and 8 of expansion and compared the exhaustion and differentiation profile with undiluted (1 × 10^6^/ml) cells. The double LAG-3/TIM-3-positive population and differentiation profile were significantly more pronounced in undiluted conditions than in all other conditions, for both the bPD1-CAR and HLA-A2 CARs, suggesting that trans-interaction is essential for the differentiation and exhaustion of both anti–HLA-A2 and anti–PD-1 CAR T cells (fig. S8).

### In vivo expansion of anti–PD-1 CAR-T cells correlates with delayed HIV rebound

**Fig. 5.**
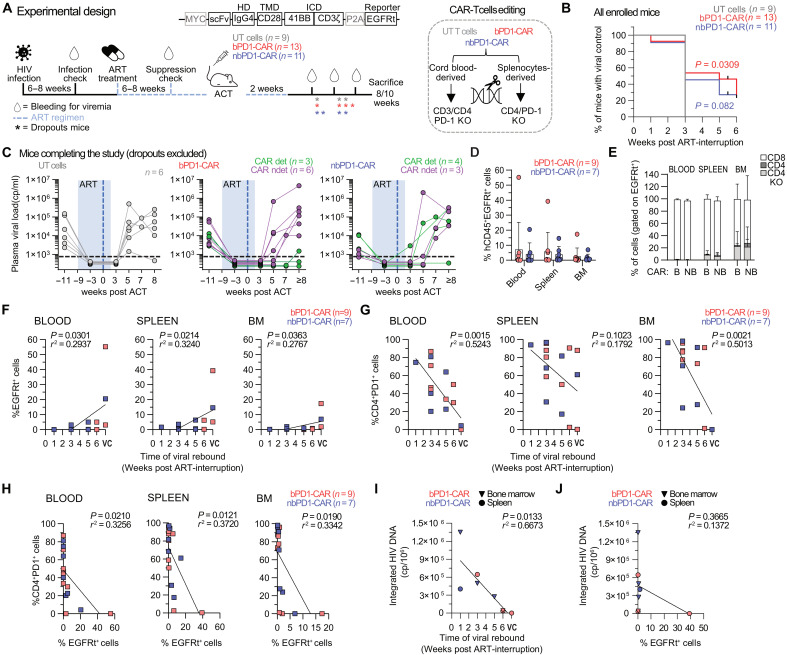
Anti–PD-1 CAR-T cells expansion in vivo correlates with depletion of CD4^+^PD-1^+^ cells and delayed viral rebound in humanized HIV-infected mice. (**A**) Experimental design. Mice received adoptive cell transfer (ACT) of anti–PD-1 CARs versus untransduced (UT) T cells under ART that was interrupted after 2 weeks (three independent experiments). The reporter used for detecting CAR-positive cells was EGFRt. (**B**) Kaplan-Meyer curve showing the percentage of mice maintaining viral control. All mice were included in the analysis. Log-rank (Mantel-Cox) test was performed using the UT control condition as reference. (**C**) Plasma viral load overtime (UT, *n* = 6; bPD1-CAR, *n* = 9; nbPD1-CAR, *n* = 7). (**D**) Percentage of CAR-T cells (EGFRt^+^ gated in CD45^+^ cells) in the spleen, BM, and blood at the time of euthanasia. Mean ± SD is shown. bPD1-CAR, *n* = 9, nbPD1-CAR, *n* = 7. (**E**) Percentage of CD8^+^, CD4^+^, and CD4^−^ CAR-T cells detected in the spleen, BM, and blood (bPD1-CAR, *n* = 3, nbPD1-CAR, *n* = 4). Mean ± SD is shown. (**F**) Correlation between the percentage of CAR-T cells (defined as CD45^+^EGFRt^+^ cells) and the time of viral rebound (bPD1-CAR, *n* = 9; nbPD1-CAR, *n* = 7). Simple linear regression was used. (**G**) Correlation between CD4^+^PD-1^+^ cells (gated in huCD45^+^EGFRt^−^ cells) and the time of viral rebound (bPD1-CAR, *n* = 9; nbPD1-CAR, *n* = 7). Simple linear regression was used. (**H**) Correlation between CD4^+^PD-1^+^ cells (gated in huCD45^+^EGFRt^−^ cells) and CAR-T cells detection (bPD1-CAR, *n* = 9; nbPD1-CAR, *n* = 7). Simple linear regression was used. (**I**) Correlation between HIV integrated DNA in CD4^+^EGFRt- sorted cells from spleen and BM and time of viral rebound (bPD1-CAR, *n* = 3; nbPD1-CAR, *n* = 3). Simple linear regression was used. (**J**) Correlation between HIV-integrated DNA in CD4^+^EGFRt^−^ sorted cells from spleen and BM and CAR-T cells detection (bPD1-CAR, *n* = 3; nbPD1-CAR, *n* = 3). Simple linear regression was used. ART, antiretroviral therapy; ACT, adoptive cell transfer; VC, viral control.

We next evaluated the ability of both anti–PD-1 CARs to control HIV replication in a hu-mice model where the leukocytes are susceptible to HIV infection (*23*–*25*). Immune reconstituted hu-mice were infected with the YU-2 HIV-1 strain from 16 weeks of age and 6 to 8 weeks later the infection was confirmed by reverse transcription polymerase chain reaction (RT-PCR). ART regimen was then initiated and once the mice suppressed, T cells were engineered either from the CD34^−^ fraction of the corresponding cord blood donors, and if the material was unavailable, from the spleen of autologous uninfected hu-mice (Fig. 5A). The quantitative analysis of PD-1^+^ cell frequency in blood and tissues was not performed before ACT. In this model, the frequency of circulating and tissue-resident human CD45 and PD-1^+^ cells can vary considerably from one control mouse to another (fig. S9).

Considering that the success of CAR-T cell therapies relies on the fitness and synergic effect of CD4^+^ and CD8^+^ T cells (*26*, *27*) and that CD4^+^ T cells are susceptible to HIV infection, we first evaluated additional editing strategies conferring HIV resistance. Thus, we tested the influence of editing the CD4, CCR5, or CXCR4 receptors and/or a combination thereof on the susceptibility to HIV infection (fig. S10, A and B). To address this issue, edited cells were exposed to CCR5 or CXCR4 tropic pseudoviral strains expressing GFP or alternatively to HIV-BaL (CCR5-tropic lab-derived HIV variant) or HIV-IIIb (CXCR4-tropic lab-derived HIV variant) replication competent viruses (fig. S10, C and D). In both cases, CD4 editing strongly reduced the susceptibility to HIV infection. Thus, we decided to target only the CD4 locus in addition to the PD-1 ± *TRBC* for T cells expanded from the CD34^−^ fraction (to prevent xenogeneic reactions). Double (CD4-PD-1) and triple (CD4-PD-1-*TRBC*) editing efficiencies were variable ranging from 50 to 95% (fig. S11).

ACT was performed under ART that was interrupted after 2 weeks and viremia was measured by quantitative PCR (qPCR) 1, 3, and 5 weeks after ART interruption (Fig. 5A).

Notably, viral rebound, defined by serum HIV level exceeding 500 HIV RNA copies/ml, which in this model and according to our experience consistently occurs between 1 and 2 weeks after ART interruption (*25*, *28*–*32*), was delayed in anti–PD-1–treated mice as compared to controls (*P* = 0.0309 for the bPD1-CAR–treated mice and *P* = 0.082 for the nbPD1-CAR) (Fig. 5B). Three mice (one nbPD1-CAR and two bPD1-CAR-treated) showed undetectable viral load until weeks 6 to 8 after ART interruption (Fig. 5C). Viral control was more efficient in the bPD1-CAR–treated group and in mice with detectable CAR-T cells in organs. Notably, CAR-T cells were detectable in the spleen, bone marrow (BM) and/or blood of 33% of bPD1-CAR–treated and 57% of the nbPD1-CAR–treated mice up to 70 days after ACT, predominantly effector memory CD8^+^ CAR-T cells regardless of the CAR origin (Fig. 5, D and E, fig. S12, and tables S1 and S2). Only few LAG-3^+^TIM-3^+^ double-positive cells were retrieved in the tissues (fig. S12). We observed a significant positive correlation between viral control and CAR-T cell detection (Fig. 5F) and negative correlations between PD-1 detection and viral control (Fig. 5G and fig. S13A) or CAR-T cells detection (Fig. 5H and fig. S13B), suggesting that the control of HIV replication was indeed conferred by anti–PD-1 CAR-T cells. To further evaluate the tissue reservoir, we have next tested the HIV DNA levels in tissues. Since CAR-T cells generated by lentiviral transduction can give false-positive results (fig. S14), we have sorted CD3^+^CD4^+^EGFRt^−^ cells from remaining frozen spleen and BM tissues. From 22 mice that completed the study, 11 spleen and eight BM samples were excluded because of insufficient material and six spleen and eight BM samples because of a lack of CD3 or *gag* detection (*n* = 11). No other mice were excluded. We found a significant correlation between integrated HIV DNA levels and time to viral rebound (Fig. 5I). There were no significant correlations between the frequency of EGFRt^+^ cells and HIV DNA levels, possibly because our analysis was underpowered (Fig. 5J).

### Anti–PD-1 CAR-T cells cause a disruption of the lymphoid architecture correlating with HIV RNA clearance

**Fig. 6.**
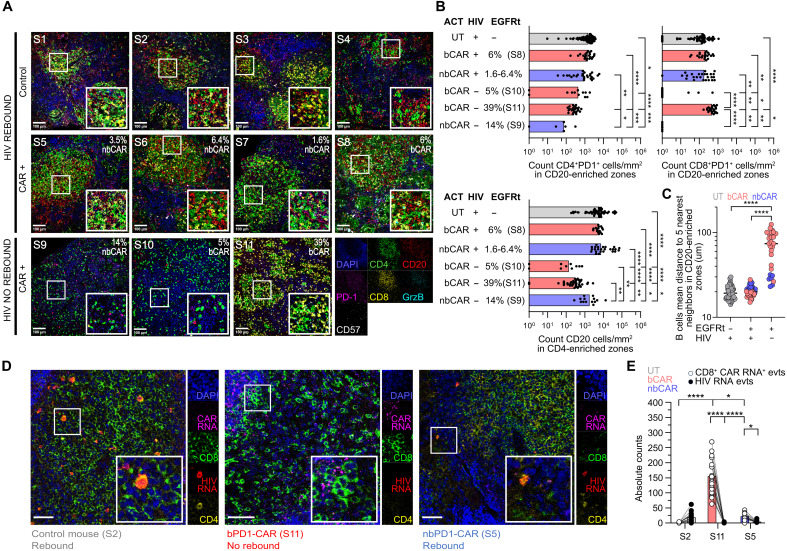
Anti–PD-1 CAR-T cells disrupt the lymphoid architecture in humanized mice spleen which correlates with HIV RNA tissue clearance. (**A**) Immunofluorescence multicolor confocal images of spleens of four control and seven treated hu-mice with detectable CAR-T cells (untransduced treated, *n* = 4; nbPD1-CAR-T treated, *n* = 4; bPD1-CAR-T treated, *n* = 3) 8 to 10 weeks after ACT. The following stainings were performed: DAPI (blue), CD4 (green), CD20 (red), PD-1 (magenta), CD8 (yellow), Grzb (cyan), and CD57 (gray). Scale bars, 100 μm. (**B**) Histo-cytometry analysis showing CD4^+^PD-1^+^, and CD8^+^PD-1^+^ cell counts/mm^2^ in the CD20-enriched areas and CD20^+^ cell counts/mm^2^ in the CD4-enriched zones. Each dot represents a CD20- or CD4-enriched region (*n* = 154 and *n* = 158). Median is shown. Kruskal-Wallis and Dunn’s multiple comparison test. All control mice (*n* = 6) and treated mice with detectable CARs (*n* = 7) that survived until the end of the experiment were included. (**C**) Mean distance between each B cell and its five nearest neighbors in the CD20-enriched regions in the 13 spleens based on IF staining data. Median is shown. Kruskal-Wallis and Dunn’s multiple comparison test was performed. (**D**) Combined IF and RNAscope for CD8 (green), DAPI (blue), HIV RNA (red), and CAR RNA (magenta) performed on the spleen of one representative control mouse (S2), one HIV suppressed responder mouse (S11), and one nonresponder mouse with viral rebound (S5) 8 weeks after ACT. Scale bars, 50 μm. (**E**) Histo-cytometry analysis showing absolute counts of CAR RNA events (gated on CD8^+^ cells) and HIV RNA events for each mouse tissue. Two-way ANOVA, Tukey’s multiple comparison test. Only statistical differences are reported as follows: *P ≤ 0.05, **P ≤ 0.01, ***P ≤ 0.001, and ****P ≤ 0.0001. S, spleen; bCAR, blocking anti–PD-1 CAR; nbCAR, nonblocking anti–PD-1 CAR; ACT, adoptive cell transfer; UT, untransduced T cells.

To better understand the mechanisms involved in the sustained control of HIV replication, we performed additional immunohistological analysis of the spleen of mice with sustained control of HIV replication (*n* = 3), mice with documented viral rebound (*n* = 4), and mice treated with untransduced T cells were used as controls (*n* = 6). The spleens of control mice exhibited peculiar lymphoid structures characterized by close interactions of B cells, CD4^+^ T cells and CD8^+^ T cells (Fig. 6A and fig. S15). Mice with sustained control of HIV replication harbored a significant reduction of CD4^+^ PD-1^+^ T cells in the CD20-enriched zones, as well as a significant depletion of CD20^+^ B cells in CD4-enriched regions as quantified by histo-cytometry (Fig. 6B and fig. S16), which correlated with a significantly lower clustering of the B cells, indicating a disruption of the lymphoid structure (Fig. 6C). For one bPD1-CAR–treated mouse, we found a strong proliferation of PD-1^+^ and Grzb^+^ CD8^+^ T cells (Fig. 6B and fig. S17).

To further confirm the control of HIV replication, we performed RNAscope studies on three animals, using one probe specific for the WPRE element of the CAR lentiviral vector and a second one for HIV RNA. We quantified both CAR-specific and HIV signals and confirmed the absence of cells harboring HIV RNA transcripts in the responder mouse S11, which is consistent with the blood qPCR results (Fig. 6, D and E, and fig. S18). In the S5 nonresponder mouse, the HIV RNA^+^ cells were mainly CD4^+^ T cells, and the proportion of HIV RNA^+^ cells among the non-CD8 CAR^+^-T cells was less than 0.4% in both treated mice (S5 and S11). This suggests that CD4 editing was sufficient to protect CAR-T cells from HIV infection (fig. S18). In conclusion, our in vitro and in vivo findings were further supported by in situ studies, collectively validating the efficacy of our CAR-T cells against HIV-infected cells.

## DISCUSSION

In this study, we demonstrate the feasibility of depleting subpopulations of human T cells expressing PD-1 in both ex-vivo cultures and hu-mice. Functionally, CAR-T cell persistence, particularly with the bPD1-CAR, correlated with HIV viral suppression, highlighting the potential of this strategy to reduce the HIV reservoir. Our in situ analysis showed a disruption of the lymphoid architecture in the white pulp area of the spleen. This finding was consistent across the two anti–PD-1 CARs despite the lower sensitivity of the nbPD1-CAR. The main advantage of such an approach is the ability to target latently infected cells, which anti-HIV CARs (e.g., CD4 based, broad neutralizing antibody based) cannot. Yet, in future studies, the synergistic effect of CAR-T cells targeting both cellular and viral antigen should be evaluated. Last, we showcase that the CAR epitope binding site modulates T cell avidity, sensitivity, and trogocytosis capacities correlating with better protection in vivo. Yet, reducing the sensitivity of CAR-T cells to PD-1 may obviate the need to delete endogenous receptor expression, thereby potentially improving manufacturability and enhancing the safety profile of the CAR-T cell product. Thus, the nbPD-1 CAR may represent an approach to mitigate off-target effects balancing therapeutic efficacy with safety considerations.

Our findings build upon prior evidence that PD-1^+^ CD4^+^ T cells serve as a key viral reservoir in lymphoid tissues (*15*). By engineering CAR-T cells to target PD-1^+^ cells, we propose an alternative approach to anti-HIV envelope-based CARs for eliminating HIV-infected cells, which remain limited by the constant mutations of the virus. This approach has also been evaluated by the group of Eichholz *et al.* (*19*), who infused anti–PD-1 CAR-T cells in SIVmac239-infected rhesus macaques. They report a selective loss of CD4^+^ PD-1^+^ T_FH_ cells correlating with suppression of SIV in the germinal centers (*19*). Yet, the nonselective nature of the anti–PD-1 CARs led to the depletion of memory CD8^+^ T cells involved in the immune surveillance against HIV (*19*, *33*, *34*). Thus, this depletion induced paradoxically an immunosuppressive state and accelerated HIV rebound in the extrafollicular reservoir upon ART interruption (*19*). This limitation may be addressed by tuning down the CAR avidity or affinity as T_FH_ express substantially higher level of PD-1 or by coinjecting HIV-specific engineered T cells. Alternatively, the specificity could be improved by generating dual receptor either specific for the HIV reservoir such as CD32a (*35*, *36*), CXCR3 (*15*), or for other surface makers highly expressed on T_FH_.

Eichholz *et al.* (*19*) showed that only half of the treated monkeys had persistent anti–PD-1 CAR-T cells, a similar percentage to our study. Despite an extensive analysis of the different confounding factors including the type of engineered T cells (CD34^−^ cord blood cells versus splenocytes), the CD45 reconstitution level, the blocking/nonblocking nature of the CAR, or the CD4 to CD8 ratios or the viral loads before ACT, we were not able to identify strong predictive biomarker (table S3). It is therefore tempting to speculate that the level of PD-1 expression within the tissue, which may not correlate with the level of reconstitution, could influence the expansion and persistence of the CAR-T cells. HIV infection, ART, and CAR-T cell proliferation in lymphopenic environments could lead to different PD-1 expression level across mice (*37*). Thus, the correlation between PD-1 abundance in tissues, CAR-T expansion, and the HIV reservoir should be addressed through a dedicated study. Suppressed HIV mice showed a selective expansion of CD8^+^ CAR-T cells, which is consistent with clinical studies demonstrating the importance of this population in predicting oncological responses (*27*, *38*). In future studies, we should evaluate the enrichment of CD7^+^CXCR3^+^ CAR-T cells before ACT as it has been recently shown to correlate with long-term CAR-T cell persistence and efficacy (*39*).

Here, we show that our anti–PD-1 CAR-T cells can disrupt the lymphoid architecture in association with a depletion of CD4^+^ PD-1^+^ cells. PD-1^high^ T_FH_ cells are essential to maintain the spatial organization of germinal centers and the follicles structure in secondary lymphoid organs (*40*, *41*). Bui *et al.* (*42*), by infusing anti-CD20 CAR-T cells in SIV-infected monkeys, efficiently ablated B cell follicles and thereby T_FH_ cells. This depletion reduced the splenic HIV reservoir, as did anti–PD-1 CAR-T cells (*42*). Furthermore, natural killer cells engineered with a fusion PD-L1 chimeric receptor depleted T_FH_ cells selectively, which inhibited B cell proliferation and antibody production (*43*). Together, these results underscore the important interplay between T_FH_ and B cells and the therapeutical potential of anti–PD-1 CAR-T cells to induce an immune reset of B cells follicles, a strategy which could be investigated in autoimmune diseases.

The present study has several limitations. First, our data showed that we were not specific to T_FH_ as we observed a depletion of CD8^+^ T cells. Moreover, infected hu-mice do not develop an HIV-specific CD8^+^ T cell response (*25*, *31*), preventing us from evaluating the respective contribution of each CAR in such depletion. The fraction of infected PD-1 cells was not explicitly quantified in HIV-infected humanized mice, which could represent another limitation of this model. Second, we have not evaluated the contribution and duration of the ART on the efficacy of the CAR-T cells, as we arbitrarily decided to maintain ART for additional 2 weeks post-ACT. Third, our study predominantly found an expansion of anti–PD-1 CAR CD8^+^ T cells in vivo. Therefore, we cannot exclude the possibility that the fitness of the anti–PD-1 CAR CD4^+^ T cells was impaired because of the gene-editing strategy, possibly reducing the efficacy of the therapy. Combining CD4^+^ and CD8^+^ CAR-T cells has been shown to have a synergistic antitumor effect in vivo (*44*, *45*). Thus, future studies should investigate the importance of coinjecting CD4^+^ CAR T cells, considering the susceptibility of CD4^+^ T cells to HIV infection. Potential alternative approaches for conferring HIV resistance include engineering peptides that inhibit the virus-cell fusion entry or deleting the CD4 receptor using safer methods, such as CRISPR-based editing (*8*). Last, even if humanized mouse models provide valuable insights into HIV latency and potential eradication strategies (*25*), they are not suitable to detect potential CAR-T cell–associated toxicities.

In summary, we have developed two anti–PD-1 CAR-T cell products that, when combined with other strategies or enhanced through engineering improvements to increase specificity, could be a step forward in achieving a cure for HIV. Thus, our approach, which targets cellular rather than viral antigens, has the major advantage of avoiding the virus’s natural capacity to mutate gp120 and escape immune surveillance. Last, our strategy could pave the way for innovative therapies for alternative indications such as autoimmune diseases considering the essential role of T_FH_ in the humoral response.

## MATERIALS AND METHODS

### Experimental design

The study aimed to develop novel anti–PD-1 CAR-T cells and evaluate their impact on the HIV reservoir in humanized mice. The experimental design included editing of human T cells using CRISPR-Cas9 technology and lentiviral transduction. The functional activity of CAR-T cells was evaluated in hu-mice susceptible to HIV infection. The dataset was supplemented with flow cytometry and histological analyses, derived from ACT experiments in hu-mice. Details on the number of biological replicates and the number of independent experiments are indicated in the figure legends and data file S1. An independent experiment includes independent isolation, manufacturing, and expansion of the primary cells. For in vivo studies, the group assignment was performed arbitrarily based on cell availability, aiming to have all groups represented (untransduced, nbPD1-CAR, and bPD1-CAR) for each donor. For in vivo data analysis, mice with less than 10% huCD45^+^ cells in the spleen were excluded. For ex vivo flow cytometry data analysis of mice, CAR detection was arbitrarily considered positive if more than 1% of huCD45^+^EGFRt^+^ events were detected.

### Human blood products

Buffy coats of deidentified human peripheral blood from healthy donors were purchased from the Swiss Transfusion Center. Density gradient centrifugation with Ficoll-Paque (Cytiva, Uppsala, Sweden, catalog no. 17144003) was used to isolate peripheral blood mononuclear cells (PBMCs).

### T cell isolation and flow cytometry

Human CD3^+^, CD4^+^, or CD8^+^ T cells were obtained using the EasySep (i) Human T Cell Enrichment Kit, (ii) CD4^+^ T Cell Isolation Kit, and (iii) CD8^+^ T Cell Enrichment Kit (StemCell Technologies, Vancouver, Canada, catalog no. 19051), respectively, following the manufacturer’s instructions from PBMCs or CD34^−^ cord blood cells. For T cells isolated from mice, splenocytes were stained with anti–CD45-FITC, anti–CD4-AF700 and anti–CD8-APC-Cy7 or PB anti-human antibodies, and CD4^+^, and CD8^+^ T cells isolated by fluorescence-activated cell (FACS) sorting on a BD FACS Aria II (BD Biosciences, Franklin Lakes, NJ, USA). For flow cytometry staining, BD LSR Fortessa Cell Analyzer (BD Biosciences, Franklin Lakes, NJ, USA) was used for sample analysis and FlowJo version 10.9.0 software (BD Biosciences, Franklin Lakes, NJ, USA) for data processing. Intracellular stainings were performed with the BD Cytofix/Cytoperm Fixation/Permeabilization Kit (BD Biosciences, Franklin Lakes, NJ, USA catalog no. 554722/23) following manufacturer’s instructions. All antibodies used in this study are listed in table S4.

### Lentivirus production

Third-generation lentiviruses were produced in human embryonic kidney 293T cells using 11.25 μg of the transgene containing vector, 2.8 μg of packaging pRSV-Rev Addgene Plasmid (catalog no. 12253) and 7.3 μg of packaging pMDLg/pRRE Addgene Plasmid (catalog no. 12251) and 3.95 μg of envelope expressing pMD2.G Addgene Plasmid (catalog no. 12259) for transfection. Third-generation lentiviral backbone was gifted by K. Scholten (Center of Experimental Therapy, Department of Oncology, University Hospital of Lausanne (CHUV), Switzerland). Sixteen hours after transfection, cells were washed, and 24 and 48 hours later, supernatant was collected and filtered with a 0.45-μm filter. Supernatant was then concentrated by ultracentrifugation at 68,000*g* for 2 hours at 12°C and stored at −80°C. The CCR5-tropic and CXCR4-tropic HIV-derived vector encoding for enhanced GFP (HIVR5GFP and HIVX4GFP) was obtained as a gift from N. Manel (Institut Curie, Paris, France) and amplified as previously described (*46*, *47*).

### Human primary T cells transduction and expansion

Human primary T cells were activated with anti-human CD3/CD28 Dynabeads (Gibco, Waltham, MA, USA, catalog no. 11131D) at 1:2 bead:T cell ratio in X-Vivo 15 medium (Lonza, Basel, Switzerland, catalog no. 02-053Q) with 5% human type AB serum from male donor (Pan-Biotech, Aidenbach, Germany, catalog no. P30-2901), 1% penicillin-streptomycin (BioConcept, Allschwill, Switzerland, catalog no. 4-01F00-H), 55 μM 2-mercaptoethanol (Gibco, Grand Island, NY, USA, catalog no. 21985-023), and 10 mM *N*-acetyl-l-cysteine (Sigma-Aldrich, Saint-Louis, MO, USA, catalog no. A9165-25G). After 18 to 20 hours from activation, lentiviral transduction was performed by adding the lentivirus of interest in the T cell culture at multiplicity of infection (MOI) = 1.4 ± 0.6. After 24 hours from lentiviral transduction cells were washed and cultured for the rest of the expansion in Roswell Park Memorial Institute (RPMI) medium (Gibco, Waltham, MA, USA, catalog no. 61870-010) supplemented with 10% fetal bovine serum (FBS; Sigma-Aldrich, Saint-Louis, MO, USA, catalog no. F7524), 1% nonessential amino acids (NEAAs; Gibco, Grand Island, NY, USA, catalog no. 11140-050), 10 mM HEPES buffer solution (Gibco, Paisley, UK, catalog no. 15630-056), 1 mM sodium pyruvate (Gibco, Waltham, MA, USA, catalog no. 11360-039), and 1% penicillin-streptomycin. Medium was supplemented with recombinant human IL-2 (30 IU/ml; rhIL-2, Miltenyi Biotec, Bergisch-Gladbach, Germany, catalog no. 130-097-746) for T cells or CD4^+^ T cells, 100 IU/ml for CD8^+^ T cells when expanded alone. For the in vivo experiments, the T cell medium was enriched with rhIL-7 (5 ng/ml; Miltenyi Biotec, Bergisch-Gladbach, Germany, catalog no. 130-095-362), rhIL-15 (5 ng/ml; Miltenyi Biotec, Bergisch-Gladbach, Germany, catalog no. 130-095-764), and IL-2 (30 IU/ml). Primary T cells were cultured at a confluency of 1 to 2 × 10^6^ cells/ml at 37°C with 5% CO_2_ and cytokines renewed every other day. On day 7 of expansion anti-CD3/CD28 beads were removed, and T cells were rested overnight in complete medium before performing functional assays. For the in vivo experiments cytokines were maintained for all duration of expansion and washed out before ACT.

### Humanized mice studies

All animal experiments were reviewed and approved by the Cantonal Veterinary Office of Zurich, Switzerland (cantonal approval number ZH176/2023 and national approval number 36332) and performed in accordance with local guidelines and Swiss animal protection law. The use of Cord blood samples was covered by KEK-StV Nr. 40/14. hu-mice were generated as previously described (*48*). In brief, newborn NOD.Cg-Prkdc^scid^ Il2rg^tm1Wjl^/SzJ (NSG) mice were irradiated with 1 gray (Gy) 1 to 3 days after birth and subsequently transplanted with 1.0 ± 0.5 × 10^5^ CD34^+^ cord blood cells via intrahepatic injection. Immune reconstitution in female and male was evaluated at 16 weeks of age by staining the peripheral blood for huCD45.

hu-mice with human engraftment level of CD45^+^ cells >5% were used for further experiments. For HIV infected mice, an intraperitoneal injection at a median tissue culture infectious dose of 2 × 10^5^ with HIV-1 YU-2 was done as previously reported (*31*). In brief, viral load was measured in the blood 6 weeks after infection using a specific qPCR assay against *vif* found in HIV-1 clade B strains. RNA was isolated from 20 μl of plasma and eluted in 60 μl of AVE buffer using the QIAamp Viral RNA Mini Kit (Qiagen, Hilden, Germany catalog no. 52904). Retrotrascription and preamplification were performed in one step using the SuperScript III Platinum One-Step qRT-PCR Kit w/ROX (Thermo Fisher Scientific, Waltham, MA, USA, catalog no. 11745100). Primer for *vif* target region are GGTCTGCATACAGGAGAAAGAG and GCTAGTTCAGGGTCTACTTGTG. PCR product was then diluted 1:10 in RNAse-free water and used for real-time qPCR with the same primers and the following probe: /56-FAM/ACTGGCATT/ZEN/TGGGTCAGGGAGTC/3IABkFQ/. For real-time qPCR, we used FastStart Essential DNA Probes Master (Roche, Roche Holding AG, Indiana, USA catalog no. 6402682001). Standards were generated by using an HIV-1 culture supernatant with a known copy number. Once infected, the mice received a combination of raltegravir, tenofovir, and emtricitabine as cART for viral load suppression.

### Mouse tissues preparation for flow cytometry and ex vivo analysis

For ex vivo analysis mice blood and spleen mononuclear cells were isolated by density gradient centrifugation using Lymphoprep (StemCell Technologies, Vancouver, Canada, catalog no. 18060), after smashing the organ on 70-μm cell strainers. For BM processing, bones were cut and flushed with a syringe, cell suspension passed through 70-μm cell strainers, and red blood cells removed using ACK lysing buffer (Lonza, Basel, Switzerland, catalog no. 10-548E). Cells were washed twice in magnetic-activated cell sorter buffer [phosphate-buffered saline (PBS) with 2 mM EDTA and 2% FBS] and stained with anti-human CD45 FITC, CD3 BV786, CD4 PE-Cy7, CD8 PB, EGFR PE, PD-1 APC, and Live/Dead APC-Cy7. Samples were fixed with 1% paraformaldehyde for 40 min at 4°C before analysis at the BD LSR Fortessa Cell Analyzer (BD Biosciences, Franklin Lakes, NJ, USA).

### Cell lines generation

The PD-1 gene in Jurkat and K562 cell lines was deleted using guides targeting the exon 2 of *PDCD1*. The crRNA sequence was shared by M. Maus, Massachusetts General Hospital, Harvard University, Boston, MA (*49*). Single cells were plated, and clones were screened for indel insertions in the *PDCD1* locus. Successful editing was confirmed by Sanger sequencing and using the ICE CRISPR Analysis Tool (Synthego, Redwood City, CA, USA) (*50*). One PD-1 KO clone for each cell line was selected to produce all other cell lines reported in the result section. To obtain differential PD-1 expression, the Jurkat PD-1 KO clone was transduced with a lentivirus encoding for PD-1 under a CD4 promoter. Single-cell clones were plated to obtain stable PD-1 expression ranging from 424 to 6047 molecules. PD-1 molecules were counted using BD Quantibrite Beads PE Fluorescence Quantitation Kit (catalog no. 340495). The design of alternative lentiviral constructs is described in the results section, and all are under an EF-1 promoter. Cell lines were routinely tested and confirmed negative for mycoplasma.

### Antibodies modeling and visualization

Model of the Fab fragments of antibodies A35795 (A35) and 135c139d6 (135c) were generated using AlphaFold (*51*, *52*). For graphical representation, the positioning of the modeled Fabs relative to the hPD-1/hPD-L1 complex [Protein Data Bank (PDB) accession no. 4ZQK] was performed using the crystal structure of the 135C–hPD-1 complex (PDB accession no. 6HIG). The positioning of A35 on the PD-1 model was performed using the HADDOCK server, ClusPro-AbEMap, and AlphaFold 3 (*53*–*55*). Visualization and structural cartoons were generated using UCSF ChimeraX (*56*).

### Antibody production and binding assay

Antibodies were produced transfecting ExpiCHO cells with 1:1 ratio of plasmid AbVec2.0-IGHG1 (Addgene Plasmid catalog no. 80795) containing the heavy chain sequence and the plasmid AbVec1.1-IGKC (Addgene Plasmid catalog no. 80796) containing the light chain sequence at the Protein Production and Structure Core Facility of the Swiss Institute of Technology in Lausanne (EPFL, Switzerland). Fabs were produced by digesting the IgG with papain using the Pierce Fab Preparation Kit (Thermo Fisher Scientific, Waltham, MA, USA, catalog no. 44985). scFv was cloned and produced as previously described (*57*).

PD-1^hi^ transgenic Jurkat cells were used to evaluate antibodies (full, Fab and scFv) binding affinities. After 30 min at 4°C, cells were washed and stained with goat anti-human IgG (H + L) cross-adsorbed secondary antibody, Alexa Fluor 568 for IgG and Fab detection (Thermo Fisher Scientific, Waltham, MA, USA, catalog no. A-21090), and a PE anti-His tag antibody for scFv detection (BioLegend, San Diego, USA catalog no. 362603). For the competitive assays, cells were incubated with the nivolumab or pembrolizumab (0.3 to 10 μg/ml) for 30 min at 4°C followed by an 30-min incubation the biotinylated anti–PD-1 Abs (clone A35 or 135C) at 2 μg/ml and 30-min incubation with BD Pharmingen PE streptavidin (BD Biosciences, Franklin Lakes, NJ, USA catalog no. 554061). Clinical lots of pembrolizumab (Keytruda, Merck) and nivolumab (Opdivo, Bristol-Myers Squibb) were obtained through the Hospitalier Universitaire Vaudois.

### Biolayer interferometry

Biolayer interferometry experiments were performed in PBS at 30°C using an Octet K2 instrument (ForteBio) as previously described (*58*). Kinetic analysis of A35/135C IgG, Fab, and scFv binding to PD-1 dimer protein was performed by biolayer interferometry immobilizing PD-1 biotinylated dimer (3 μg/ml) on streptavidin biosensors (Sartorius, Göttingen, Germany, catalog no. 18-5019) dipped into a solution of the Fab/scFv at different concentrations diluted in PBS (ranging from 40 to 240 nM), and the nm shift was recorded on the Octet. Analysis was performed using the Octet software with 1.1 analyte fitting for the interaction with Fabs and scFvs.

### RNP formulation and electroporation protocols

Cas9 protein was synthetized by the Protein Production and Structure Core Facility of EPFL (Switzerland). CRISPR-Cas9 guides were reconstituted as previously described (*59*, *60*). In brief, lyophilized crRNAs were resuspended in IDT nuclease-free duplex buffer at a 160 μM final concentration and stored at −80°C. Guides were reconstituted by mixing crRNA and tracrRNA at 1:1 ratio and incubated at 37°C for 30 min for annealing. Then, 45 μM recombinant Cas9 was added to the 80 μM RNA at a 1:1 ratio and incubated at 37°C for 15 min. Ribonucleoproteins (RNPs) were kept at 4°C up to 2 weeks. Table S5 provides a list of the sequences of the guides used in this study. Human primary T cells were electroporated on day 0 before activation using the program EH-115 in the Lonza 4D 96-well electroporation system. After electroporation, cells were recovered with prewarmed complete medium and rested for 10 min at 37°C.

### In vitro functional assays

Functional assays were performed on day 8 of expansion after overnight resting without cytokines. For luciferase-based killing assay, 0.02 × 10^6^ Jurkat cells were cocultured with the CAR-T cells at different E:T ratios. After 24 hours, luciferase assay reading was performed as previously described (*61*). In brief, cells were lysed for 20 min by shaking at 300 rpm with 50 μl of harvesting buffer [50 mM 2-morpholinoethanesulfonic acid sodium (NaMES, pH 7.8, Sigma-Aldrich, Saint-Louis, MO, USA, catalog no. 1061970100), 50 mM tris-HCl (pH 7.8), and 1 mM dithiothreitol (Thermo Fisher Scientific, Waltham, MA, USA, catalog no. R0861) + 0.2% Triton X-100 (Thermo Fisher Scientific, Waltham, MA, USA, catalog no. 9002-93-1) in distilled water]. Then, 50 μl of luciferase assay buffer [125 mM MES, 125 tris-HCl (pH 7.8), 25 mM magnesium acetate tetrahydrate (Sigma-Aldrich, Saint-Louis, MO, USA, catalog no. M0631), and 2.5 mM adenosine triphosphate (Thermo Fisher Scientific, Waltham, MA, USA, catalog no. R0441) in distilled water] was added for 1 minute. Thereafter, 50 μl of luciferin buffer [1 mM d-luciferin (Thermo Fisher Scientific, Waltham, USA, catalog no. 88292) in 5 mM potassium dihydrogen phosphate (Merck, Darmstadt, Germany, catalog no. 1.04873] was added to the lysate. Luminescence was measured on a Synergy H1 Hybrid reader (BioTek, Winooski, VT, USA).

For the activation assay, 0.1 × 10^6^ CAR-T cells were cocultured with 120-Gy-irradiated K562 cell line at 1:1 E:T ratio. CD25 and CD71 levels were assessed 48 hours later by flow cytometry. For cytokine production assessment, GolgiPlug (BD Biosciences, Franklin Lakes, NJ, USA, catalog no. 555029) was added in culture after overnight incubation and phorbol 12-myristate acetate (25 ng/ml; Sigma-Aldrich, Saint-Louis, MO, USA, catalog no. P1585) and 1 μM ionomycin (Sigma-Aldrich, Saint-Louis, MO, USA, catalog no. I9657) were added to positive control conditions 14 hours before readout. After 44 hours from the start of the coculture, intracellular staining was performed to quantify IFN-γ, TNF-α, IL-2, perforin, and Grzb production.

### HIV virus

HIV-1 BaL and IIIb strains were obtained from the National Institutes of Health (NIH) HIV Reagent Program. HIV-YU2 strain for hu-mice infection was purchased from the Centre for AIDS Reagents (National Institute for Biological Standards and Control, UK catalog no. 100 840).

### HIV integrated DNA

CD3^+^CD4^+^EGFRt^−^ T cells were FACS sorted from remaining spleen and BM frozen cells. Cells were lysed using lysis buffer [10 mM tris-HCl (pH 8.0), 50 nM KCl, and proteinase K (400 mg/ml), Thermo Fisher Scientific, Waltham, MA, USA, catalog no. 25530049] and integrated HIV DNA and CD3 gene copy numbers were quantified using a cross-clade nested Alu PCR, as previously described (*46*, *62*). Only samples with detectable CD3 and *gag* copies were included in the analysis. The frequency of integrated HIV DNA copies per million cells was calculated as previously described (*46*, *62*).

### In vitro HIV-1 infection assays

Primary CD4^+^ T cells were isolated and edited for CD4/CCR5/CXCR4 in all different combinations of single, double or triple KO. After 7 days of expansion, edited T cells were restimulated with anti-CD3 mAb plate bound and 3 days later infected with replication incompetent (R5 or X4 tropic) GFP^+^ HIV. Infection rate was evaluated by flow cytometry 4 days later. For the virological assay, edited T cells were sorted on day eight before restimulation. Cells were then infected on day 11 with replication competent HIV strains, HIV-BaL (R5 tropic), or HIV-1 IIIB Strain (X4 tropic). After 7 days in culture HIV RNA was quantified in the supernatant.

### Mouse tissue preparation and staining for immunofluorescence

Half of the mice spleen was harvested for histological analyses. Tissues were fixed in 10% formalin overnight and then stored in 1%formalin-PBS. Three-micrometer sections from formalin-fixed paraffin-embedded blocks were prepared. Deparaffinization, antigen retrieval, and fluorescent staining procedures were performed on the Ventana Discovery Ultra Autostainer (Roche Diagnostics, Basel, Switzerland) as previously described (*63*). In brief, staining procedure consisted of subsequent cycles of antibody incubations and blocking steps using the Opal blocking/antibody diluent solutions (Akoya Biosciences, Marlborough, MA, USA, catalog no. ARD1001EA). Primary antibody incubation was 32 min followed by a 16-min incubation with secondary horseradish peroxidase–labeled antibodies and a final one with optimized fluorescent Opal tyramide signal amplification dyes (Opal 7-color Automation IHC kit and Opal650 reagent pack, Akoya Biosciences, catalog nos. NEL821001KT and FP1496001KT, respectively). Repeated antibody denaturation cycles were performed. Tissue sections were washed and stained with Spectral 4′,6-diamidino-2-phenylindole (DAPI) from Akoya Biosciences for 4 min, rinsed in water with soap and mounted using DAKO mounting medium (Agilent, Santa Clara, CA, USA, catalog no. S302380-2). Details on the antibodies, clones, and dilutions are listed in table S6.

### Quantitative imaging analysis (histo-cytometry)

Confocal images were quantitatively analyzed through histo-cytometry procedure as previously described (*64*, *65*), using Imaris software version 9.9.0 (Bitplane). Shortly, three-dimensional segmented surfaces (based on the nuclear signal) were generated with the Surface Creation module of Imaris. Quantitative data generated this way, containing values like average intensities, together with volume and sphericity of the surfaces were exported in Microsoft Excel format. Files were then converted to comma separated value (CVS) files and imported into FlowJo (version 10) for further analysis. For quantification of the different cell subsets manual gating of CD20-enriched or CD4-enriched regions was performed on the basis of cell density.

### Quantitative spatial analysis of B cells organization

To assess the spatial distribution of B cells within tissue sections, we performed a nearest-neighbor distance analysis using single-cell spatial coordinates. The (*X*, *Y*) spatial coordinates of CD20^+^B cells were extracted for each enriched region, with at least 20 evts, and exported into separate Excel files, with each file corresponding to a specific region. Multiple regions were analyzed from each donor. Cells annotated with “KM_label = 1” indicated the CD20^+^ B cell population and were retained for further downstream analysis. The data were then imported and processed into R (version 4.4.2) using the “readxl” and “dplyr” packages. The spatial coordinates were transformed into a planar point pattern object using the ppp() function from the “spatstat” package, with tissue boundaries defined by the coordinate ranges specified through owin(). To evaluate the local spatial structure, we calculated the Euclidean distance from each CD20^+^ B cell to its five nearest neighbors (*k* = 5) using the nndist() function. The analysis returned a set of distance values corresponding to the five nearest neighbors for each cell. To quantify the overall spatial organization within each donor, we computed the mean of the nearest-neighbor distance values across all regions analyzed per donor.

### RNAscope

RNAscope in situ hybridization for the CAR RNA and HIV RNA visualization was performed according to the manufacturer’s instructions using the RNAscope multiplex fluorescent detection kit version 2 (Advanced Cell Diagnostics, ACD, Newark, CA, USA catalog no. 323110). After deparaffinization on the Ventana Discovery Ultra Autostainer (Roche Diagnostics, Basel, Switzerland) as described above, the sections were treated with RNAscope Hydrogen Peroxide for 10 min at room temperature (RT), followed by an antigen retrieval step at 100°C for 15 min. Subsequently, sections were incubated with Protease III for 15 min at 40°C in a HybEz hybridization oven (ACD) to permeabilize the tissue. To detect CAR-T cells, we used the RNAscope Probe- WPRE-O4-C2 (Advanced Cell Diagnostics, ACD, Newark, CA, USA catalog no. 540341-C2) recognizing the Woodchuck hepatitis virus posttranscriptional regulatory element specifically present in the 3′ untranslated region of the CAR lentivector. For HIV RNA detection we used RNAscope Probe – V-HIV1-CladeB-O1-C1 (Advanced Cell Diagnostics, ACD, Newark, CA, USA catalog no. 1120101-C1). To be able to visualize the RNA signals, we used the tyramide-based detection system by Akoya (Opal 7-color Automation IHC kit, Akoya Biosciences, catalog no. NEL821001KT). Afterward, slides were incubated in antibody blocking for 30 min and then the protein staining was performed. Applied antibodies were verified for their compatibility with RNAscope protocol. Samples were incubated for 90 min, RT, with conjugated anti-CD4 AF700 and anti-CD8 A647. The samples were then counterstained with DAPI, and mounting was performed thereafter as described above.

### Image acquisition

Acquisition of the images was performed on a Leica Stellaris 8 SP8 confocal system running the LAS-X software, at 512 × 512 pixel density for overview of the full tissue and 1024 × 1024 for high-resolution image using the 0.75× optical zoom and a 20× objective (40× objective for RNAscope). Leica LAS-AF Channel Dye Separation module (Leica Microsystems) was used to create and apply a compensation matrix. For RNAscope, histocytometry analysis the whole tissue was divided into 18 to 25 squares for a systematic analysis of the number of CAR^+^ RNA and HIV^+^ RNA cells across the tissue.

### Statistical analysis

For most in vitro experiments with human primary cells, each replicate was a unique healthy donor. Depending on material availability and cells count numbers, cells from one donor were transduced with different MOIs and were considered as independent replicates. For in vivo experiments, each replicate was a single mouse. GraphPad Prism 10 software was used for all statistical analysis. Data distribution was assessed using the Shapiro-Wilk test. For normally distributed data, statistical differences between two groups were determined using two-tailed parametric Student’s *t* tests, whereas comparisons among three or more groups were conducted using one-way or two-way analysis of variance (ANOVA) with Tukey’s post hoc correction when comparing all groups between them or Dunnett’s post hoc correction when comparing all groups to control group. For data that did not pass the normality test, statistical differences between two groups were determined using unpaired *t* test, while statistical differences between three or more groups were performed using Kruskal-Wallis tests with Dunn’s post hoc analysis. Correlations were analyzed with a simple linear regression model. Kaplan-Meyer survival curves for viral load were analyzed with log-rank (Mantel-Cox) statistical test.

Specific test and statistical method are described for each analysis in the figure legend. Statistical significance values of the stars are the following: *P ≤ 0.05, **P ≤ 0.01, ***P ≤ 0.001, and ****P ≤ 0.0001. Only statistical differences are reported. No outliers were excluded.
