# Supplementary material for: Targeting PD-1+ T cells with chimeric antigen receptors to reduce the HIV reservoir
Source: Sci Adv. 2026 Apr 24;12(17):eaeb7602. doi: 10.1126/sciadv.aeb7602 (PMC13108567; doi:10.1126/sciadv.aeb7602)
Supplement: Supplementary file 1 — Figs. S1 to S18 Tables S1 to S6 Legend for data file S1 [file sciadv.aeb7602_sm.pdf]

Science Advances
AAAS

# Supplementary Materials for

## Targeting PD-1$^+$ T cells with chimeric antigen receptors to reduce the HIV reservoir

Laura Ermellino *et al.*

Corresponding author: Yannick D. Muller, yannick.muller@chuv.ch

*Sci. Adv.* **12**, eaeb7602 (2026)
DOI: 10.1126/sciadv.aeb7602

**The PDF file includes:**

Figs. S1 to S18
Tables S1 to S6
Legend for data file S1

**Other Supplementary Material for this manuscript includes the following:**

Data file S1

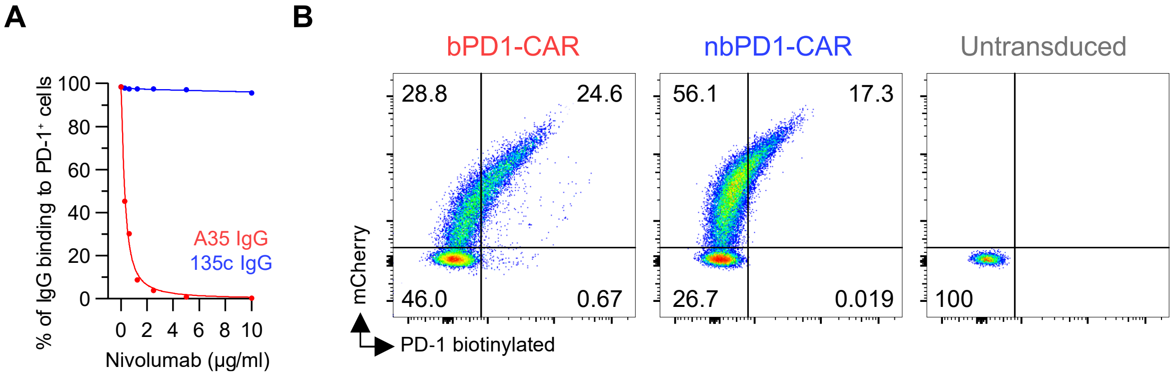


**Fig. S1. Competitive binding assay and PD-1 binding to the CAR. (A)** Competitive binding assay with Nivolumab. Symbols are means of two independent experiments. **(B)** Representative flow cytometry showing the binding of a PD-1 biotinylated protein (0.3µg/ml) to the anti-PD-1 CAR or untransduced T cells. Abbreviations. IgG, Immunoglobulin G. PD-1, Programmed cell death protein 1. CAR, Chimeric Antigen Receptor. bPD1-CAR, blocking anti-PD-1 CAR. nbPD1-CAR, non-blocking anti-PD-1 CAR.

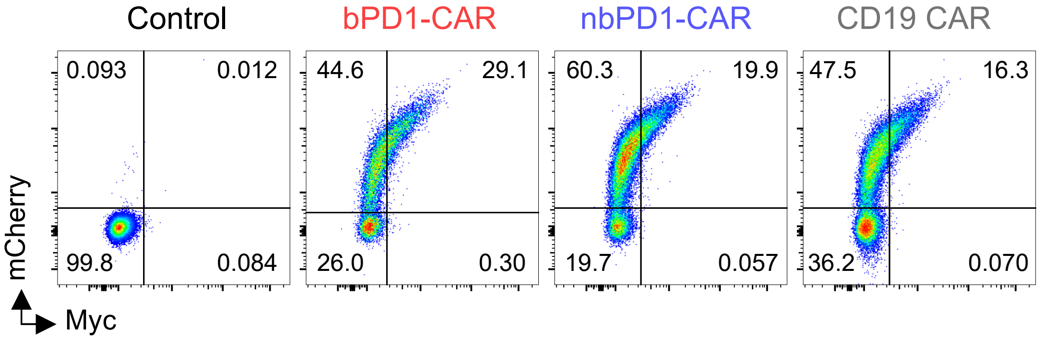


**Fig. S2. CAR expression and transduction level.** Representative flow cytometry data showing mCherry and Myc co-expression on CAR-T cells. Abbreviations. CAR, Chimeric Antigen Receptor. bPD1-CAR, blocking anti-PD-1 CAR. nbPD1-CAR, non-blocking anti-PD-1 CAR.

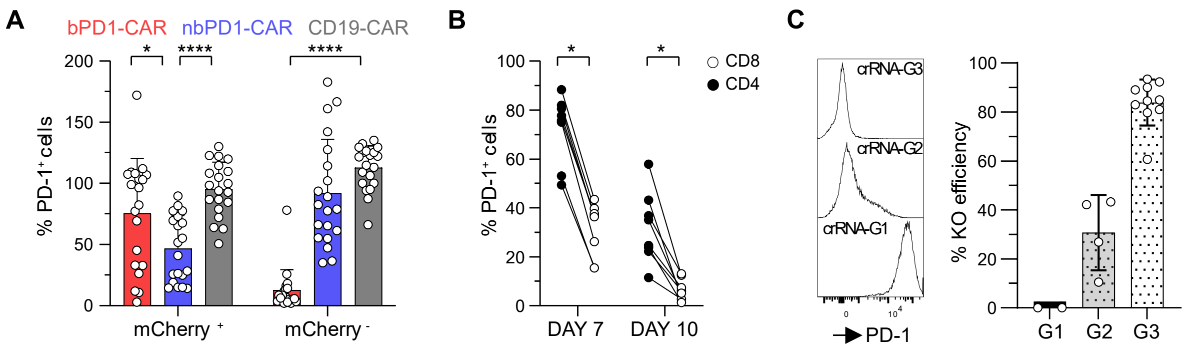


**Fig. S3. PD-1 expression in CAR-T cells and PD-1 knock-out efficacy**. **(A)** PD-1 expression in Cherry positive versus Cherry negative populations. Mean ± SD values of 8 donors, 8 independent experiments, 2-3 internal replicates transduced with different MOIs are shown. Two-way ANOVA, Tukey's multiple comparison test. **(B)** PD-1 expression in polyclonal $CD4^+$ and $CD8^+$ unedited T cells on day 7 and 10 of expansion. Mean of 8 donors, 8 independent experiments is shown. Multiple Paired T test. **(C)** PD-1 editing tests with three different crRNAs and their efficiency (Mean ± SD values of n=2-10). Only statistical differences are reported as follows: * $P \leq 0.05$, ** $P \leq 0.01$, *** $P \leq 0.001$, **** $P \leq 0.0001$. Abbreviations. PD-1, Programmed cell death protein 1. CAR, Chimeric Antigen Receptor. bPD1-CAR, blocking anti-PD-1 CAR. nbPD1-CAR, non-blocking anti-PD-1 CAR. KO, knock out. crRNA, CRISPR RNA.

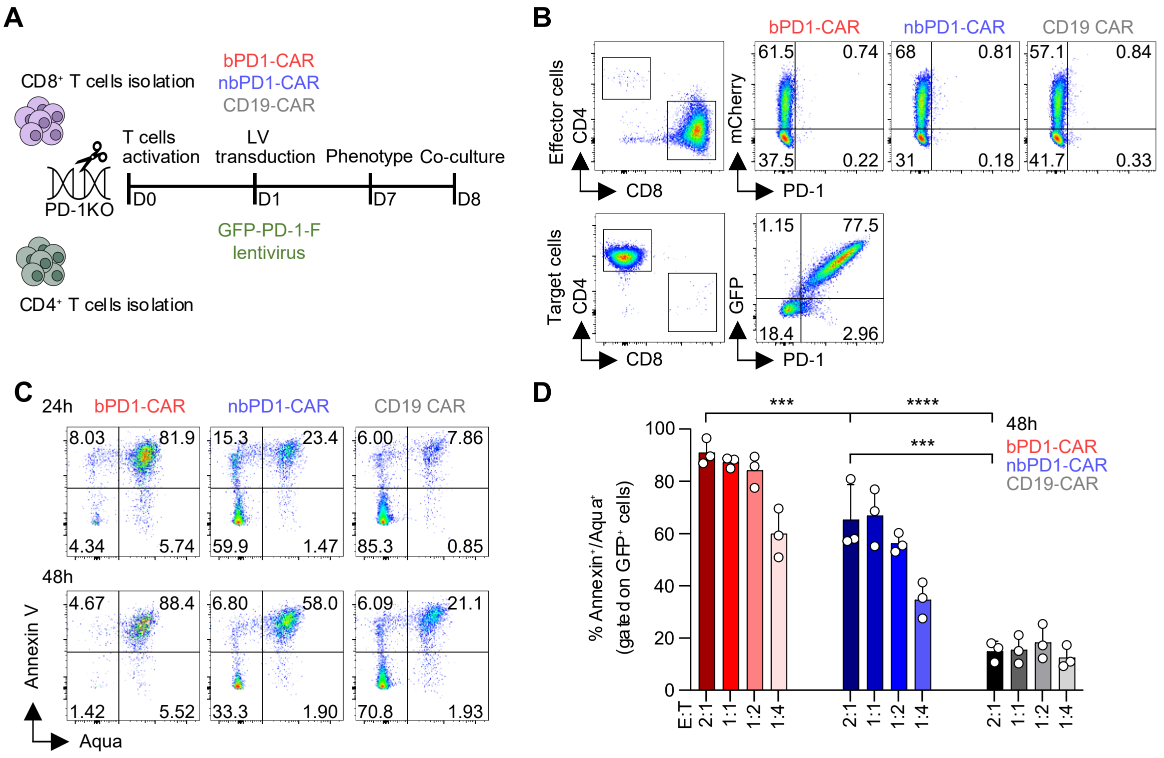


**Fig. S4. *In vitro* killing assay. (A)** Experimental design. CD8$^+$ and CD4$^+$ T cells were isolated, edited for PD-1, activated and expanded separately. CD8$^+$ T cells were transduced with different CAR constructs, while CD4 cells were transduced to stably express a PD-1-GFP-F fusion protein. Killing assay was performed on day 8. **(B)** Representative flow cytometry of the phenotype of the target and effector cells used in the assay. **(C)** Representative flow cytometry showing Annexin/Aqua staining in CD4$^+$GFP$^+$ target cells after 24h and 48h co-culture at 1:1 ration with CARs. **(D)** Cumulative percentage of Annexin/Aqua$^+$ CD4$^+$PD-1$^+$ target cells in a FACS-based killing assay after 48h. Mean ± SD of 3 donors and 3 independent experiments is shown. Two-way ANOVA, Tukey's multiple comparison test. Only statistical differences are reported as follows: * $P \leq 0.05$, ** $P \leq 0.01$, *** $P \leq 0.001$, **** $P \leq 0.0001$. Abbreviations. PD-1, Programmed cell death protein 1. CAR, Chimeric Antigen Receptor. bPD1-CAR, blocking anti-PD-1 CAR. nbPD1-CAR, non-blocking anti-PD-1 CAR. LV, lentivirus. KO, knock out.

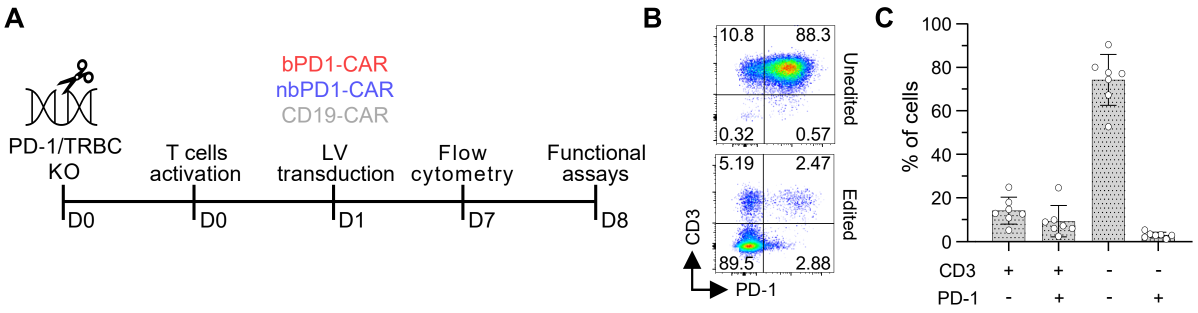


**Fig. S5. Experimental timeline and editing efficiency for functional assays. (A)** Experimental timeline of CAR T cells generation for functional assays. **(B)** Representative flow cytometry staining showing CD3 and PD-1 on day 7. **(C)** Cumulative data of editing efficiencies showing CD3 and PD-1. The Mean ± SD values of 7 donors is shown. <u>Abbreviations</u>. CAR, Chimeric Antigen Receptor. bPD1-CAR, blocking anti-PD-1 CAR. nbPD1-CAR, non-blocking anti-PD-1 CAR. LV, lentivirus. TRBC, TCR beta chain. KO, knock out.

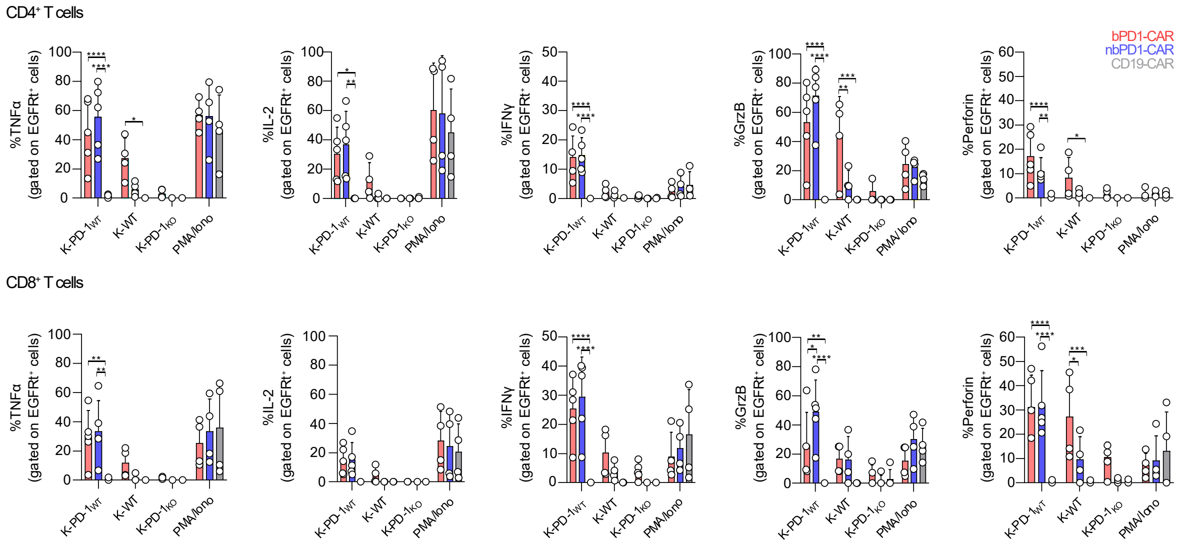


**Fig. S6. Effector molecules secretion by anti-PD-1 CAR-T cells.** Frequencies of TNF-α, IL-2, IFN-γ, Granzyme B and perforin expression in CAR-T cells after a 48h co-culture with K-PD-$1_{WT}$, K-PD-$1_{KO}$ or K-WT. Percentage values were obtained by subtracting the value of the corresponding unstimulated condition. Mean ± SD values of 3-5 donors and 5 independent experiments are shown. Two-way ANOVA, Tukey's multiple comparison test. Only comparisons among different CARs in each condition were performed. Only statistical differences are reported as follows: * $P \leq 0.05$, ** $P \leq 0.01$, *** $P \leq 0.001$, **** $P \leq 0.0001$.

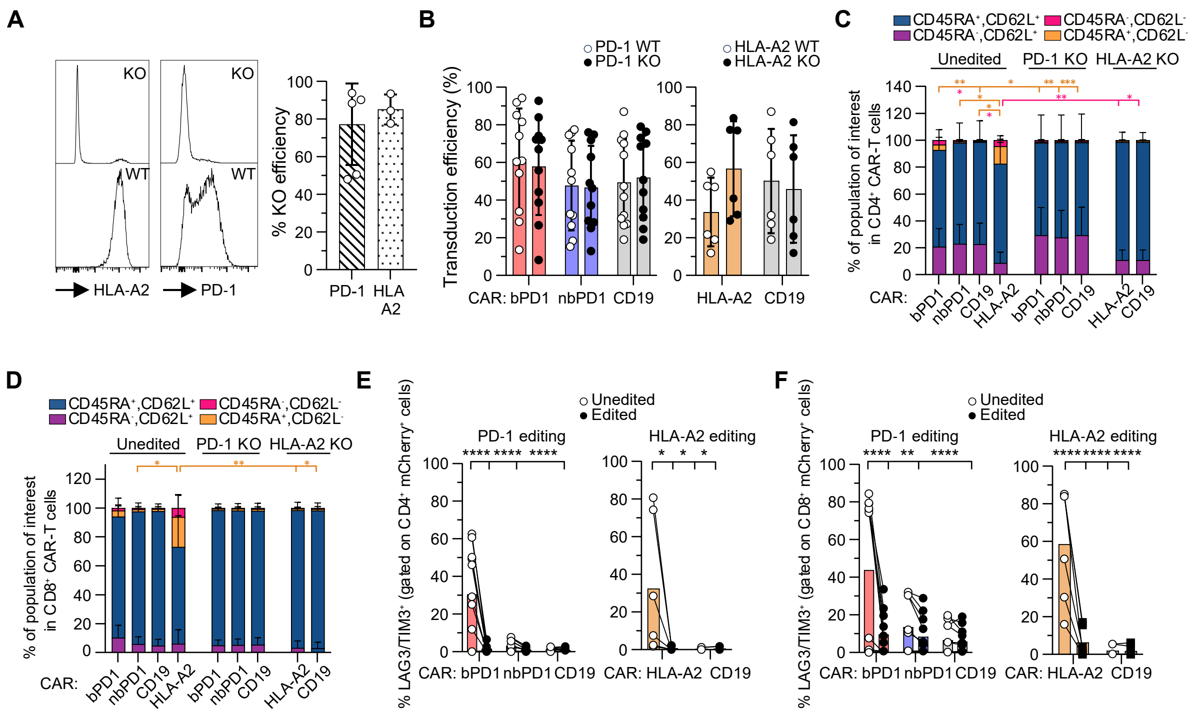

**Fig. S7. PD-1 and HLA-A2 editing and CAR-T cells differentiation and exhaustion profile. (A)** Representative flow cytometry staining showing PD-1 and HLA-A2 expression +/- editing (left). Cumulative data (right). For PD-1 KO, mean ± SD values of the untransduced condition of 5 donors in 5 independent experiments is shown. For the HLA-A2 KO mean ± SD values of the untransduced condition of 3 donors in 3 independent experiments is shown. **(B)** Cumulative data showing the transduction efficiency +/- editing. Mean ± SD values of 3-5 donors in 3-5 independent experiments with 1-2 internal replicates (different MOI transduction: 1.4 ± 0.6) is shown. Two-way ANOVA, Tukey's multiple comparison test. **(C-D)** Cumulative data showing CD62L and CD45RA expression in edited versus unedited $CD4^+$ (C) and $CD8^+$ (D) CAR-T cells. The mean ± SD values of 3-5 donors in 3-5 independent experiments with 1-2 internal replicates (different MOI transduction: 1.4 ± 0.6) is shown. Kruskal-Wallis and Dunn's multiple comparison test was performed. **(E-F)** Percentage of $LAG3^+TIM3^+$ double positive $CD4^+$ (E) and $CD8^+$ (F) T cells in unedited versus edited CAR-T cells. Paired values corresponding 3-5 donors in 3-5 independent experiments with 1-2 internal replicates (different MOI transduction: 1.4 ± 0.6) are shown. Two-way ANOVA, Tukey's multiple comparison test. Only statistical differences are reported as follows: * $P \leq 0.05$, ** $P \leq 0.01$, *** $P \leq 0.001$, **** $P \leq 0.0001$. Abbreviations. CAR, Chimeric Antigen Receptor. bPD1-CAR, blocking anti-PD-1 CAR. nbPD1-CAR, non-blocking anti-PD-1 CAR. KO, knock out.

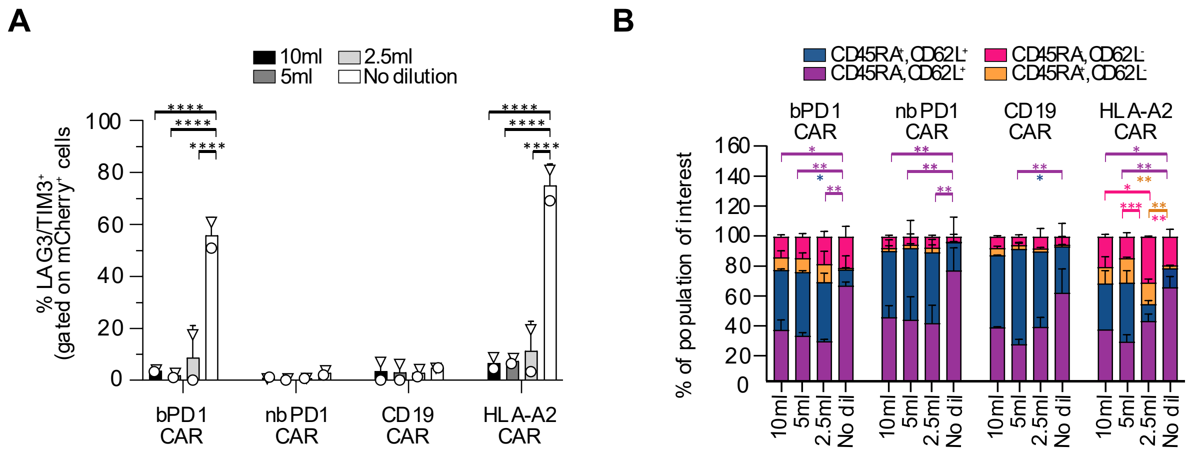


**Fig. S8. CAR trans-interactions as a main driver of exhaustion and differentiation. (A)** Percentage of LAG3$^+$TIM3$^+$ double positive CAR-T cells in different dilutions. Paired values corresponding to 2 donors in 2 independent experiments are shown. Two-way ANOVA, Tukey's multiple comparison test. Only comparisons among the different conditions for each CAR are shown. **(B)** Cumulative data showing CD62L and CD45RA expression in CAR-T cells in the different dilutions conditions. The mean ± SD values of 2 donors in 2 independent experiments is shown. Two-way ANOVA, Tukey's multiple comparison test. Only comparisons among the different conditions for each CAR are shown. Only statistical differences are reported as follows: * P ≤ 0.05, ** P ≤ 0.01, *** P ≤ 0.001, **** P ≤ 0.0001.

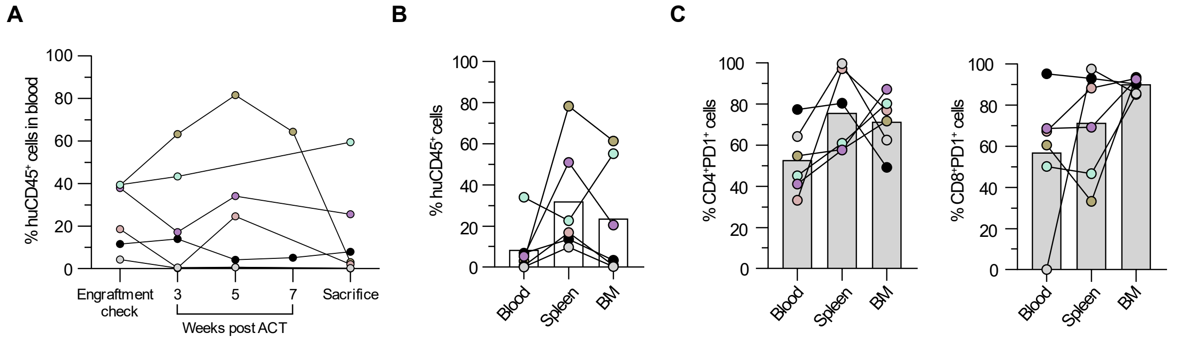


**Fig. S9. HuCD45$^{+}$ cells in blood and tissues. (A)** Percentage of huCD45$^{+}$ cells in blood overtime in 6 control mice (UT, n=6) and **(B)** within the spleen and bone marrow tissues at sacrifice point. **(C)** Percentage of CD4$^{+}$PD1$^{+}$ and CD8$^{+}$PD1$^{+}$ cells in 6 control mice (UT, n=6) in blood and within the spleen and bone marrow tissues at sacrifice point. Abbreviations. ACT, adoptive cell transfer. BM, bone marrow.

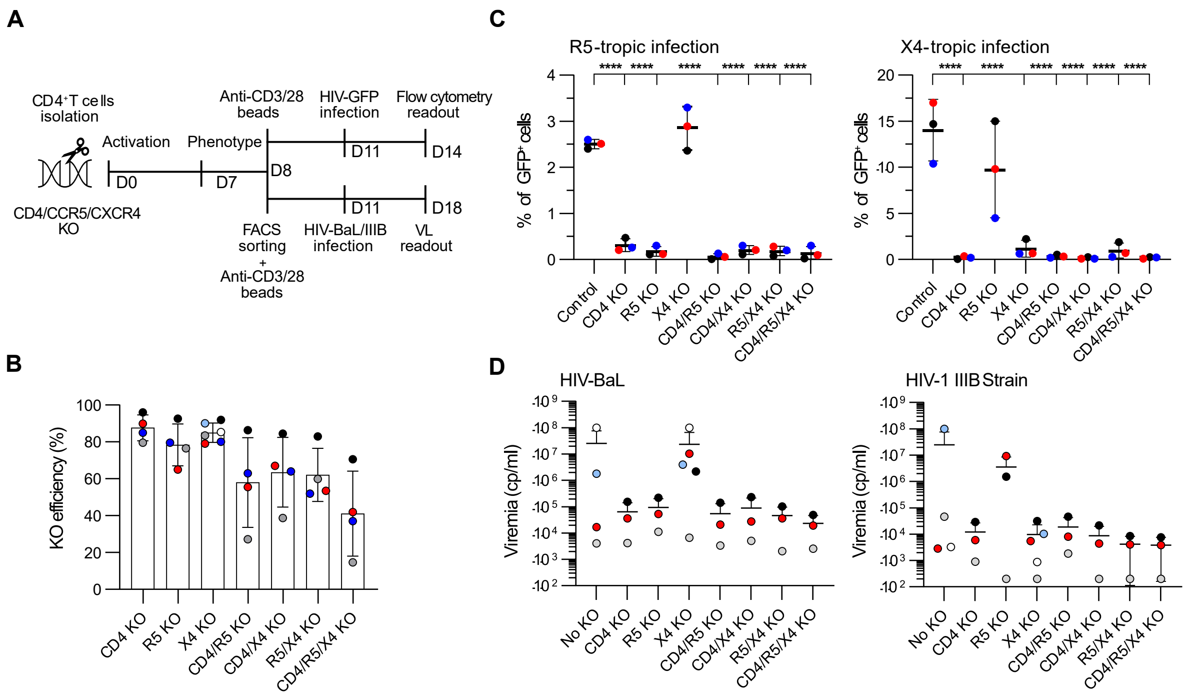


**Fig. S10. Generation of HIV resistant CD4$^{+}$ T cells. (A)** Experimental timeline. **(B)** KO efficiency for CD4, CCR5 and CXCR4 receptors in different combinations in CD4 primary T cells. Mean ± SD of 4-6 donors in 5 independent experiments. **(C)** Percentage of GFP$^{+}$ infected cells from a R5 tropic strain on the left and a X4 tropic strain on the right for each edited population. Mean ± SD values of 3 donors in 3 independent experiments. Comparison of each edited population with the unedited control is shown. Ordinary one-way ANOVA, Dunnett's multiple comparisons test was performed. **(D)** Viral load (cp/ml) expressed in log scale measured in supernatant of CD4$^{+}$ edited T cells 7 days after infection with HIV-BaL strain on the left or HIV-1 IIIB strain of the right. Mean ± SD values of 3-5 donors in 4 independent experiments. Comparison of each edited population with the unedited control is shown. Ordinary one-way ANOVA, Dunnett's multiple comparisons test was performed. Only statistical differences are reported as follows: * $P \leq 0.05$, ** $P \leq 0.01$, *** $P \leq 0.001$, **** $P \leq 0.0001$. Abbreviations. HIV, Human Immunodeficiency virus. KO, knock out. R5, CCR5. X4, CXCR4. CAR, Chimeric Antigen Receptor. bPD1-CAR, blocking anti-PD-1 CAR. nbPD1-CAR, non-blocking anti-PD-1 CAR. VL, viral load. ACT, adoptive cell transfer. ART, antiretroviral therapy. BM, bone marrow.

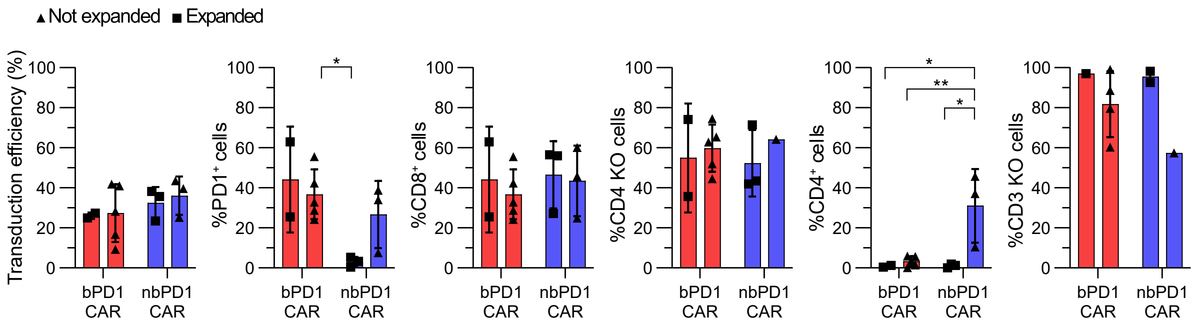


**Fig. S11. CAR-T cells phenotype before *in vivo* infusion.** Phenotype of CAR-T cells before injection in HIV infected hu-mice comparing the ones that expanded *in vivo* versus the ones that did not. Mean ± SD of 3 to 5 donors is shown. Two-way ANOVA, Tukey's multiple comparisons test. Only statistical differences are reported as follows: * $P \leq 0.05$, ** $P \leq 0.01$, *** $P \leq 0.001$, **** $P \leq 0.0001$. Abbreviations. KO, knock out. CAR, Chimeric Antigen Receptor. bPD1-CAR, blocking anti-PD-1 CAR. nbPD1-CAR, non-blocking anti-PD-1 CAR.

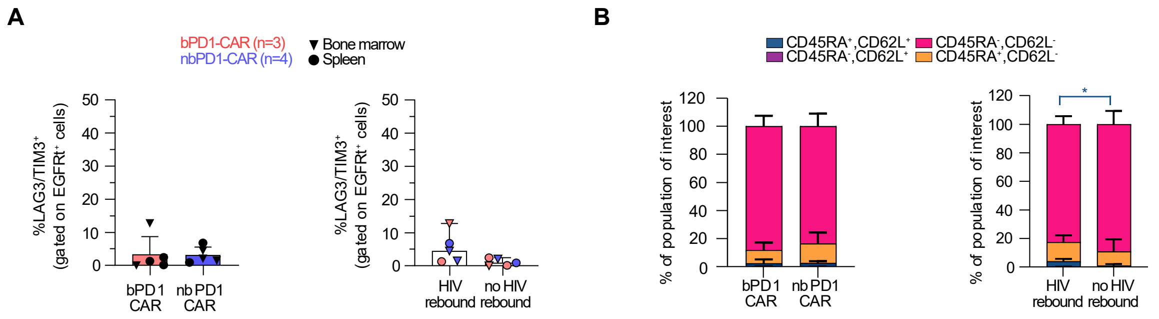


**Fig. S12.** ***In vivo*** **CAR-T cells phenotype**. **(A)** Cumulative percentage of LAG3$^+$TIM3$^+$ double positive CAR-T cells in spleen and bone marrow of mice with detectable EGFRt, split by CAR (bPD1-CAR, n=3, nbPD1-CAR, n=4) or as responders. **(B)** Cumulative percentage of CD62L and CD45RA expression in CAR-T cells in spleen and bone marrow of mice with detectable EGFRt, split by CAR (bPD1-CAR, n=3, nbPD1-CAR, n=4) or as responders. Mean ± SD values are shown. Unpaired T test was performed. Only statistical differences are reported as follows: * $P \leq 0.05$, ** $P \leq 0.01$, *** $P \leq 0.001$, **** $P \leq 0.0001$.

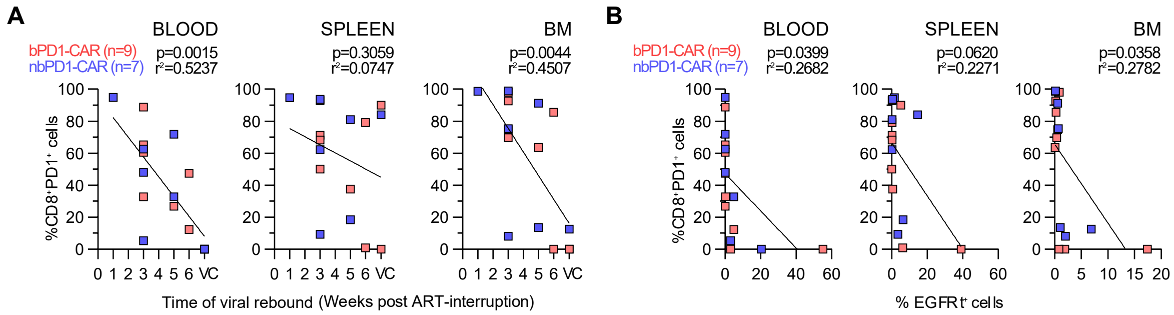


**Fig. S13. Correlations between the number of CD8$^{+}$PD-1$^{+}$ cells, viral rebound and CAR-T detection. (A)** Correlation between CD8$^{+}$PD-1$^{+}$ cells (gated in huCD45$^{+}$EGFRt$^{-}$ cells) and the time of viral rebound (bPD1-CAR, n=9, nbPD1-CAR, n=7). Simple linear regression was used. **(B)** Correlation between CD8$^{+}$PD-1$^{+}$ cells (gated in huCD45$^{+}$EGFRt$^{-}$ cells) and CAR-T cells detection (bPD1-CAR, n=9, nbPD1-CAR, n=7). Simple linear regression was used. Abbreviations. CAR, Chimeric Antigen Receptor. bPD1-CAR, blocking anti-PD-1 CAR. nbPD1-CAR, non-blocking anti-PD-1 CAR. VC, viral control. EGFRt, Truncated epidermal growth factor receptor. ART, antiretroviral therapy. BM, bone marrow.

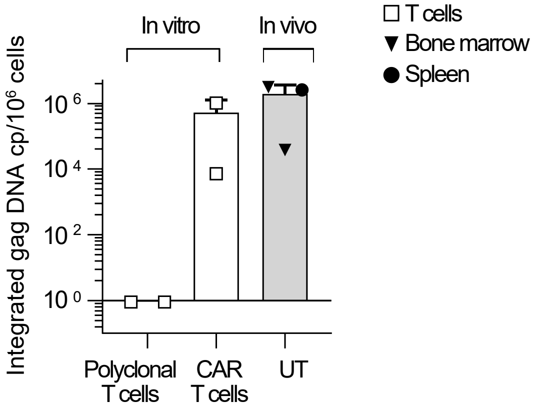


**Fig. S14. Gag integrated DNA copies in controls.** Integrated gag DNA copies per million of $CD3^{+}CD4^{+}$ sorted cells from (empty square) expanded polyclonal T cells versus CAR-T cells (two technical replicates) from healthy donors and $CD3^{+}CD4^{+}$ sorted cells from two control HIV infected mice (sorted from the spleen (plain circle) or bone marrow (plain triangle)) treated with polyclonal untransduced (UT) T cells. Abbreviations. UT, untransduced.

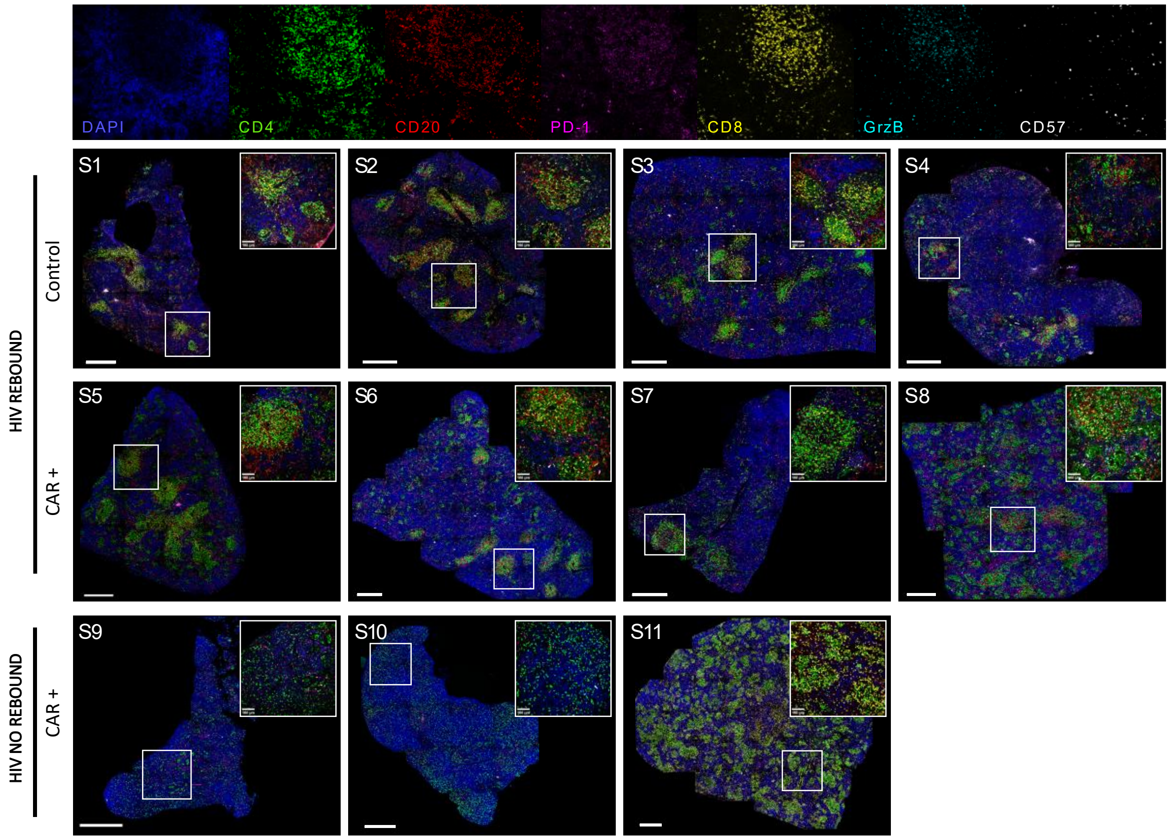


**Fig. S15. Confocal images of full tissue sections of spleens from humanized mice**. Overview images of the full spleen sections acquired at the confocal microscope (20x magnification). Areas selected for higher resolution images of figure 6 are shown. Immunofluorescence multicolour panel showing DAPI (blue), CD4 (green), CD20 (red), PD-1 (magenta), CD8 (yellow), GrzB (cyan) and CD57 (gray). Scale bar, 500 µm. Abbreviations. HIV, Human Immunodeficiency virus. CAR, Chimeric Antigen Receptor.

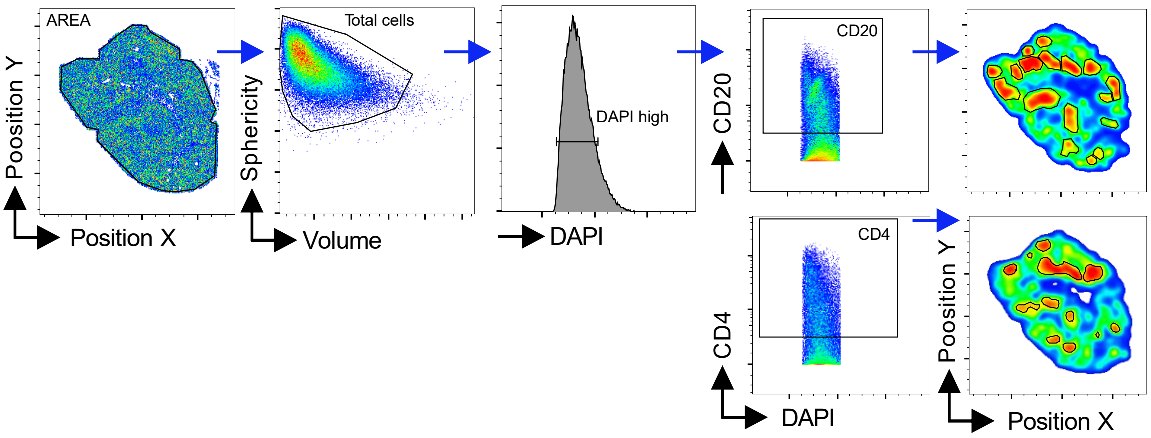


**Fig. S16. Gating strategy for histo-cytometry analysis.** Gating strategy for histo-cytometry analysis of CD20 or CD4-enriched zones, based on cell density.

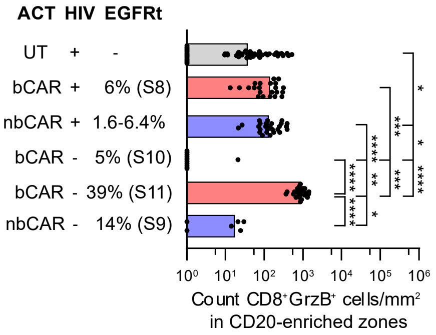


**Fig. S17. Histo-cytometry quantification of $CD8^+GrzB^+$ cells in CD20-enriched zones**. Histo-cytometry analysis showing $CD8^+$ $GrzB^+$ cell counts/$mm^2$ in CD20-enriched zones. Each dot represents a CD20-enriched zone (n=155). All 13 mice shown in Fig.6B are included. Median is shown. Kruskal-Wallis test and Dunn's test. Only statistical differences are reported as follows: * P ≤ 0.05, ** P ≤ 0.01, *** P ≤ 0.001, **** P ≤ 0.0001. Abbreviations. bCAR, blocking anti-PD-1 CAR. nbCAR, non-blocking anti-PD-1 CAR. GrzB, Granzyme B. EGFRt, Truncated epidermal growth factor receptor. UT, untransduced T cells.

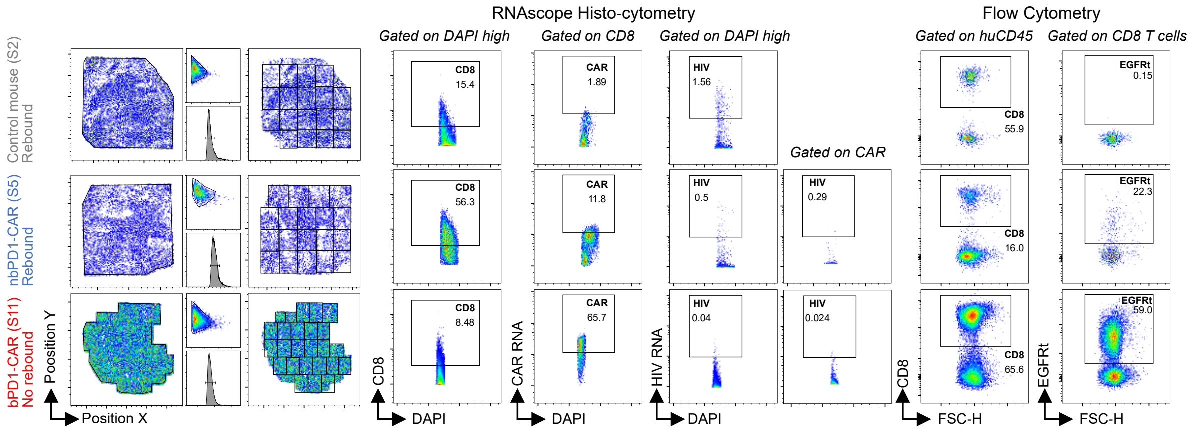


**Fig. S18. RNAscope histo-cytometry gating strategy**. Gating strategy for histo-cytometry analysis of RNAscope data (left). RNAscope histo-cytometry and flow cytometry comparison of mice S2, S5, S11 (right). HIV RNA$^+$ cells gated on DAPI, CAR$^+$ total T cells and CAR$^+$ non-CD8 T cells. Abbreviations. bCAR, blocking anti-PD-1 CAR. nbCAR, non-blocking anti-PD-1 CAR. GrzB, Granzyme B. EGFRt, Truncated epidermal growth factor receptor.

**Table S1.** Viremia levels pre- and post- CAR-T cells treatment, mice death rates and huCD45 and EGFRt flow cytometry levels in organs.

| **Viremia before ART** | **MOUSE ID** | blood (1 week post ART interruption - 3 | blood (3 weeks post ART interruption - 5 | blood (5 weeks post ART interruption - 7 | blood (6/8 weeks post ART interruption - 8/10 weeks post ACT) | EGFR detection in organs (% of huCD45) | | | % of huCD45+ cells in organs | | |
|---|---|---|---|---|---|---|---|---|---|---|---|
| UT T cells | | | | | | SPLEEN | BM | BLOOD | SPLEEN | BM | BLOOD |
| 1.22E+05 | 5629 | NEG | 5.03E+05 | *missing* | 1.02E+04 | | | | 20.4 | 0.66 | 2.04 |
| 1.46E+04 | 5630 | NEG | 2.76E+04 | *dead* | *dead* | | | | | | |
| 7.83E+03 | 5660 | NEG | 7.87E+03 | *missing* | 5.42E+04 | | | | 59 | 18 | 25.5 |
| 1.50E+05 | 5674 | NEG | 1.43E+03 | *missing* | 4.58E+02 | | | | 11.8 | 0.1 | 0.1 |
| 3.87E+03 | 5659 | NEG | 2.69E+04 | *missing* | 2.55E+05 | | | | 26.4 | 47.3 | 59.5 |
| 7.12E+04 | 5693 | NEG | 1.21E+04 | 7.11E+04 | *dead* | | | | | | |
| 1.77E+04 | 5720 | NEG | 7.16E+04 | 2.02E+05 | *dead* | | | | | | |
| 2.21E+04 | 5727 | NEG | 5.23E+04 | 2.96E+04 | 2.45E+04 | | | | 24 | 3.99 | 7.98 |
| 2.29E+03 | 5730 | NEG | 1.97E+04 | 8.78E+04 | 1.34E+05 | | | | 85.2 | 71.7 | 2.99 |
| bPD1-CAR | | | | | | | | | | | |
| 8.97E+03 | 5672 | 1.47E+03 | *dead* | *dead* | *dead* | | | | | | |
| 3.87E+03 | 5641 | NEG | 1.27E+04 | *missing* | 4.26E+04 | 0.19 | 0.21 | 0.23 | 40.6 | 4.11 | 9.48 |
| 7.22E+04 | 5657 | NEG | 1.51E+04 | *missing* | 3.08E+05 | 0.19 | 0.87 | 0.00 | 25.3 | 1.91 | 4.23 |
| 5.30E+04 | 5658 | NEG | 1.57E+03 | *missing* | 1.88E+05 | 0.24 | 0.41 | 0.04 | 74.9 | 35.6 | 30.1 |
| 5.85E+04 | 5722 | NEG | 4.45E+03 | *dead* | *dead* | | | | | | |
| 1.08E+04 | 5723 | NEG | NEG | NEG | *dead* | | | | | | |
| 1.07E+05 | 5750 | NEG | NEG | NEG | *dead* | | | | | | |
| 7.09E+03 | 5754 | NEG | NEG | 1.04E+04 | 9.72E+03 | 0.59 | 0.087 | 0.17 | 22.2 | 74 | 30.9 |
| 8.91E+03 | 5675 | NEG | *missing* | *missing* | NEG | 5.19 | 17.41 | 3.12 | 13.6 | 0.71 | 1.06 |
| 1.27E+04 | 5733 | NEG | NEG | NEG | NEG | 39.2 | 1.92 | 55.2 | 39.8 | 2.09 | 79.8 |
| 2.81E+03 | 5734 | NEG | NEG | NEG | 1.49E+03 | 6.24 | 0.81 | 5 | 64.3 | 52.4 | 30.6 |
| 3.14E+03 | 5882 | NEG | 6.69E+05 | *missing* | 4.77E+06 | 0.178 | 0.59 | 0.027 | 52.3 | 48 | 1.94 |
| 3.24E+03 | 5886 | NEG | NEG | *missing* | 1.15E+05 | 0.218 | 0.3 | 0.012 | 49.9 | 49 | 25.4 |
| nbPD1-CAR | | | | | | | | | | | |
| 1.62E+05 | 5669 | NEG | 1.40E+03 | *dead* | *dead* | | | | | | |
| 7.18E+02 | 5644 | NEG | NEG | 7.30E+03 | 3.34E+04 | 0.19 | 0.56 | 0.06 | 64.2 | 18.9 | 22.7 |
| 1.24E+04 | 5645 | NEG | 6.20E+02 | *dead* | *dead* | | | | | | |
| 1.73E+03 | 5681 | NEG | 7.96E+02 | 2.57E+04 | 3.15E+05 | 0.54 | 0.18 | 0.00 | 35.1 | 17 | 5.43 |
| 1.10E+03 | 5724 | NEG | NEG | NEG | *dead* | | | | | | |
| 1.12E+04 | 5736 | NEG | 4.73E+04 | 1.01E+05 | 6.02E+04 | 3.51 | 2.05 | 3.1 | 39.1 | 26.9 | 29 |
| 9.95E+02 | 5737 | NEG | NEG | 2.41E+04 | 3.54E+04 | 6.4 | 1.06 | 5.01 | 35.6 | 41.5 | 26.7 |
| 1.02E+04 | 5679 | 2.83E+03 | *missing* | *missing* | 3.03E+05 | 1.58 | 0.14 | 0.00 | 70.7 | 10.4 | 4.87 |
| 1.94E+03 | 5676 | NEG | *missing* | *missing* | NEG | 14.57 | 6.90 | 20.50 | 22.4 | 61.3 | 51.2 |
| 1.05E+03 | 5888 | NEG | 2.01E+06 | *missing* | 1.90E+05 | 0.189 | 0.669 | 0.026 | 54.2 | 47.4 | 15.3 |
| 1.80E+03 | 5885 | NEG | NEG | *missing* | *dead* | | | | | | |

**Table S2.** Subgrouping of the mice in CAR detectable and non-detectable groups based on 1% threshold of huCD45$^{+}$EGFRt$^{+}$ cells.

EGFRt Flow Cytometry frequencies

CAR detectable

bPD1-CAR

| MOUSE | SPLEEN | BM | BLOOD |
|---|---|---|---|
| 5675 | 5.19 | 17.41 | 3.12 |
| 5733 | 39.2 | 1.92 | 55.2 |
| 5734 | 6.24 | 0.81 | 5 |

nbPD1-CAR

| MOUSE | SPLEEN | BM | BLOOD |
|---|---|---|---|
| 5676 | 14.57 | 6.90 | 20.50 |
| 5679 | 1.58 | 0.14 | 0.00 |
| 5736 | 3.51 | 2.05 | 3.1 |
| 5737 | 6.4 | 1.06 | 5.01 |

CAR non detectable

bPD1-CAR

| MOUSE | SPLEEN | BM | BLOOD |
|---|---|---|---|
| 5754 | 0.59 | 0.087 | 0.17 |
| 5641 | 0.19 | 0.21 | 0.23 |
| 5657 | 0.19 | 0.87 | 0.00 |
| 5658 | 0.24 | 0.41 | 0.04 |
| 5882 | 0.178 | 0.59 | 0.027 |
| 5886 | 0.218 | 0.3 | 0.012 |

nbPD1-CAR

| MOUSE | SPLEEN | BM | BLOOD |
|---|---|---|---|
| 5644 | 0.19 | 0.56 | 0.06 |
| 5681 | 0.54 | 0.18 | 0.00 |
| 5888 | 0.189 | 0.669 | 0.026 |

**Table S3.** Tables show all different factors involved in CAR-T cells generation and ACT, comparing the mice groups based on viral rebound or CAR detection.

| | **no HIV rebound** **N =3**[1] | **HIV rebound** **N = 13**[1] | **p-value**[2] |
|---|---|---|---|
| **Mice with detectable CAR** | 3 / 3 (100%) | 4 / 13 (31%) | 0.063 |
| **Mouse sex** | | | **0.036** |
| F | 3 / 3 (100%) | 3 / 13 (23%) | |
| M | 0 / 3 (0%) | 10 / 13 (77%) | |
| **Mice cohort** | | | >0.9 |
| 1 | 2 / 3 (67%) | 6 / 13 (46%) | |
| 2 | **1 / 3 (33%)** | 4 / 13 (31%) | |
| 3 | 0 / 3 (0%) | 3 / 13 (23%) | |
| **Mouse donor** | | | 0.5 |
| T874 | 0 / 3 (0%) | 1 / 13 (7.7%) | |
| T875 | 0 / 3 (0%) | 1 / 13 (7.7%) | |
| HFL CD34+ Dec 2016 | 0 / 3 (0%) | 1 / 13 (7.7%) | |
| HFL CD34+ Oct2020 | 0 / 3 (0%) | 1 / 13 (7.7%) | |
| T787 | 0 / 3 (0%) | 1 / 13 (7.7%) | |
| T794 | 2 / 3 (67%) | 0 / 13 (0%) | |
| T814 | 0 / 3 (0%) | 2 / 13 (15%) | |
| T849 | 1 / 3 (33%) | 3 / 13 (23%) | |
| T874 | 0 / 3 (0%) | 1 / 13 (7.7%) | |
| U320 | 0 / 3 (0%) | 2 / 13 (15%) | |
| **Cells origin** | | | >0.9 |
| CD34- | 2 / 3 (67%) | 7 / 13 (54%) | |
| spleen | 1 / 3 (33%) | 6 / 13 (46%) | |
| **Type of CAR** | | | >0.9 |
| bPD1 | 2 / 3 (67%) | 7 / 13 (54%) | |
| nbPD1 | 1 / 3 (33%) | 6 / 13 (46%) | |
| **Number of total injected cells (MIO)** | 3.20 [1.96, 8.00] | 4.00 [3.00, 8.00] | 0.5 |
| **Number of injected CAR T cells** | 1.20 [0.55, 2.00] | 1.00 [0.80, 1.50] | >0.9 |
| **CRISPR editing** | | | 0.8 |
| CD3/CD4/PD1 KO | 0 / 3 (0%) | 4 / 13 (31%) | |
| CD4/CD3/PD1 | 2 / 3 (67%) | 3 / 13 (23%) | |
| CD4/PD1 | 1 / 3 (33%) | 4 / 13 (31%) | |
| Unedited | 0 / 3 (0%) | 2 / 13 (15%) | |
| **% CD4 T cells** | 1 [0, 1] | 3 [1, 9] | 0.092 |
| **% CD4 KO T cells** | 72 [36, 74] | 45 [42, 68] | 0.4 |
| **% CD8 T cells** | 27 [26, 63] | 46 [24, 56] | >0.9 |
| **Ratio CD8/CD4** | 0.37 [0.34, 1.70] | 1.00 [0.32, 1.27] | >0.9 |
| **State of cells at ACT** | | | 0.2 |
| fresh | 1 / 3 (33%) | 10 / 13 (77%) | |
| frozen | 2 / 3 (67%) | 3 / 13 (23%) | |
| **Duration of expansion (days)** | 12.00 [11.00, 12.00] | 11.00 [11.00, 11.00] | 0.2 |
| **Engraftment before ACT (%huCD45)** | 23 [23, 64] | 29 [20, 52] | 0.8 |
| **Plasma VL before ACT** | 8,910 [1,940, 12,700] | 3,240 [1,730, 10,200] | 0.6 |
| **Plasma VL at sacrifice** | 0 [0, 0] | [illegible]5,000 [35,400, 303,0[illegible] | **0.010** |
| **Engraftment at sacrifice (%huCD45 blood)** | 51 [1, 80] | 23 [5, 29] | 0.4 |

[1] n / N (%); Median [IQR]
[2] Fisher's exact test; Wilcoxon rank sum test; Wilcoxon rank sum exact test

| | **no detectable CAR** **N = 9**[1] | **detectable CAR** **N = 7**[1] | **p-value**[2] |
|---|---|---|---|
| **HIV rebound** | 9 / 9 (100%) | 4 / 7 (57%) | 0.063 |
| **Mouse sex** | | | 0.3 |
| F | 2 / 9 (22%) | 4 / 7 (57%) | |
| M | 7 / 9 (78%) | 3 / 7 (43%) | |
| **Mice cohort** | | | 0.14 |
| 1 | 5 / 9 (56%) | 3 / 7 (43%) | |
| 2 | 1 / 9 (11%) | 4 / 7 (57%) | |
| 3 | 3 / 9 (33%) | 0 / 7 (0%) | |
| **Mouse donor** | | | **0.044** |
| T874 | 1 / 9 (11%) | 0 / 7 (0%) | |
| T875 | 1 / 9 (11%) | 0 / 7 (0%) | |
| HFL CD34+ Dec 2016 | 1 / 9 (11%) | 0 / 7 (0%) | |
| HFL CD34+ Oct2020 | 1 / 9 (11%) | 0 / 7 (0%) | |
| T787 | 1 / 9 (11%) | 0 / 7 (0%) | |
| T794 | 0 / 9 (0%) | 2 / 7 (29%) | |
| T814 | 1 / 9 (11%) | 1 / 7 (14%) | |
| T849 | 0 / 9 (0%) | 4 / 7 (57%) | |
| T874 | 1 / 9 (11%) | 0 / 7 (0%) | |
| U320 | 2 / 9 (22%) | 0 / 7 (0%) | |
| **Cells origin** | | | 0.6 |
| CD34- | 6 / 9 (67%) | 3 / 7 (43%) | |
| spleen | 3 / 9 (33%) | 4 / 7 (57%) | |
| **Type of CAR** | | | 0.6 |
| bPD1 | 6 / 9 (67%) | 3 / 7 (43%) | |
| nbPD1 | 3 / 9 (33%) | 4 / 7 (57%) | |
| **Number of total injected cells (MIO)** | 3.90 [3.00, 4.50] | 8.00 [3.20, 8.00] | 0.4 |
| **Number of injected CAR T cells** | 0.90 [0.80, 1.00] | 2.00 [1.20, 2.00] | **0.040** |
| **CRISPR editing** | | | 0.056 |
| CD3/CD4/PD1 KO | 4 / 9 (44%) | 0 / 7 (0%) | |
| CD4/CD3/PD1 | 2 / 9 (22%) | 3 / 7 (43%) | |
| CD4/PD1 | 1 / 9 (11%) | 4 / 7 (57%) | |
| Unedited | 2 / 9 (22%) | 0 / 7 (0%) | |
| **% CD4 T cells** | 9 [3, 12] | 1 [0, 2] | **0.020** |
| **% CD4 KO T cells** | 63 [45, 70] | 42 [36, 72] | 0.6 |
| **% CD8 T cells** | 33 [22, 46] | 56 [27, 63] | 0.056 |
| **Ratio CD8/CD4** | 0.50 [0.28, 1.00] | 1.27 [0.37, 1.70] | 0.056 |
| **State of cells at ACT** | | | 0.6 |
| fresh | 7 / 9 (78%) | 4 / 7 (57%) | |
| frozen | 2 / 9 (22%) | 3 / 7 (43%) | |
| **Duration of expansion (days)** | 11.00 [11.00, 11.00] | 11.00 [11.00, 12.00] | 0.4 |
| **Engraftment before ACT (%huCD45)** | 36 [16, 60] | 26 [23, 39] | >0.9 |
| **Plasma VL before ACT** | 3,240 [1,730, 7,090] | 8,910 [1,940, 11,200] | 0.8 |
| **Plasma VL at sacrifice** | [illegible]8,000 [42,600, 308,00[illegible] | 1,490 [0, 60,200] | **0.034** |
| **Engraftment at sacrifice (%huCD45 blood)** | 15 [5, 25] | 29 [5, 51] | 0.3 |

[1] n / N (%); Median [IQR]
[2] Fisher's exact test; Wilcoxon rank sum test; Wilcoxon rank sum exact test

**Table S4.** Antibodies used for Flow Cytometry.

| Target | Clone | Fluorophore | Vendor | Catalog number | Country |
|---|---|---|---|---|---|
| CD3 | UCHT-1 | PE-Cy7 | BD Biosciences | 563423 | Franklin Lakes, NJ, USA |
| CD3 | OKT3 | BV785 | BioLegend | 317330 | San Diego, CA, USA |
| CD4 | RPA-T4 | AF700 | BD Biosciences | 557922 | Franklin Lakes, NJ, USA |
| CD4 | RPA-T4 | FITC | BD Biosciences | 555346 | Franklin Lakes, NJ, USA |
| CD4 | SK3 | BUV395 | BD Biosciences | 563550 | Franklin Lakes, NJ, USA |
| CD4 | RPA-T4 | PE-Cy7 | BioLegend | 300512 | San Diego, CA, USA |
| CD8 | SK1 | BV421 | BioLegend | 344748 | San Diego, CA, USA |
| CD8 | RPA-T8 | APC-Cy7 | BD Biosciences | 557760 | Franklin Lakes, NJ, USA |
| CD8 | SK1 | PE | BD Biosciences | 345773 | Franklin Lakes, NJ, USA |
| CD25 | CD25-4E3 | APC | Thermo Fisher Scientific | 17-0257-42 | Waltham, MA, USA |
| CD71 | CY1G4 | APC-Cy7 | BioLegend | 334110 | San Diego, CA, USA |
| CD71 | M-A712 | FITC | BD Biosciences | 555536 | San Diego, CA, USA |
| CTV | CellTrace Violet | Pacific Blue | Invitrogen | C34557 | Waltham, MA, USA |
| CD45 | H130 | FITC | BioLegend | 304006 | San Diego, CA, USA |
| CD45RA | HI100 | BV650 | BD Biosciences | 563963 | Franklin Lakes, NJ, USA |
| CD62L | DREG-56 | BV-421 | BD Biosciences | 563862 | Franklin Lakes, NJ, USA |
| LAG3 | 11C3C65 | PerCP/Cyanine5.5 | BioLegend | 369312 | San Diego, CA, USA |
| TIM3 | CF38-2E2 | BV 785 | BioLegend | 345032 | San Diego, CA, USA |
| PD-1 | EH12.2H7 | APC | BioLegend | 329908 | San Diego, CA, USA |
| PD-1 | EH12.1 | PE-Cy7 | BD Biosciences | 561272 | Franklin Lakes, NJ, USA |
| IFN-γ | B27 (RUO) | PE-Cy7 | BD Biosciences | 557643 | Franklin Lakes, NJ, USA |
| TNF-α | MAb11 | FITC | BioLegend | 502906 | San Diego, CA, USA |
| IL-2 | MQ1-17H12 | PerCP-Cy5.5 | BioLegend | 500322 | San Diego, CA, USA |
| Perforin | B-D48 | PE | BioLegend | 353304 | San Diego, CA, USA |
| Granzyme B | GB11 | Alexa Fluor 647 | BD Biosciences | 560212 | Franklin Lakes, NJ, USA |
| CCR5 | REA245 | APC | Miltenyi Biotec GmbH | 130-120-057 | Miltenyi Biotec, Bergisch-Gladbach, Germany |
| CXCR4 | 12G5 | PE-Cy7 | BioLegend | 306514 | San Diego, CA, USA |
| EGFR | AY13 | PE | BioLegend | 352904 | San Diego, CA, USA |
| EGFR | AY13 | FITC | BioLegend | 352908 | San Diego, CA, USA |
| DAPI | Live/Dead | NA | Invitrogen | D1306 | Waltham, MA, USA |
| Anti-his tag | J095G46 | PE | BioLegend | 362603 | San Diego, CA, USA |
| Streptavidin | | PE | BD Biosciences | 554061 | Franklin Lakes, NJ, USA |
| Goat anti-Human IgG (H+L) Cross-Adsorbed Secondary antibody | | Alexa Fluor 568 | Invitrogen | A-21090 | Waltham, MA, USA |
| LIVE/DEAD® Fixable Aqua Dead Cell Stain Kit | | NA | Thermo Fisher Scientific | L34957 | Waltham, MA, USA |
| Zombie NIR Fixable Viability Kit | | NA | BioLegend | 423105/06 | San Diego, CA, USA |

**Table S5.** crRNA sequences.

| Name | Sequence |
|---|---|
| TRBC | CCCACCAGCTCAGCTCCACG |
| CD4 | GGCAAGGCCACAATGAACCG |
| PD-1 | CTGCAGCTTCTCCAACACAT |
| HLA-A2 | CCTCGTCCTGCTACTCTCGG |
| CCR5 | CAATGTGTCAACTCTTGACA |
| CXCR4 | CACTTCAGATAACTACACCG |

**Table S6.** Antibodies used for immunofluorescence staining of spleen tissues.

| Target | Clone | Dilution | Catalogue Number | Fluorophore | Vendor |
|---|---|---|---|---|---|
| CD4 | EPR6855 | 1/300 | ab133616 | | Abcam |
| PD1 | NAT105 | 1/100 | 3137 | | Bio Optica |
| CD57 | NK-1 | 1/200 | Mob163 | | CliniSciences |
| CD20 | L26 | 1/400 | NCL-L-CD20-L26 | | Leica system |
| CD8 | C8/144b | 1/50 | M7103 | | Agilent |
| GrzB | GrB-7 | 1/40 | MON7029C | | Monosan |
| | | | | | |
| CD4 | Polyclonal | 1/35 | FAB8165N | AF 700 | Bio-techne |
| CD8 | C8/144B | 1/25 | 372906 | AF 647 | Biolegend |

**Data file S1 (separate file).** Individual-level data for all experiments.
